# Antigenic Variation of East/Central/South African and Asian Chikungunya Virus Genotypes in Neutralization by Immune Sera

**DOI:** 10.1371/journal.pntd.0004960

**Published:** 2016-08-29

**Authors:** Chong-Long Chua, I-Ching Sam, Andres Merits, Yoke-Fun Chan

**Affiliations:** 1 Department of Medical Microbiology, Faculty of Medicine, University of Malaya, Kuala Lumpur, Malaysia; 2 Institute of Technology, University of Tartu, Tartu, Estonia; The University of Pittsburgh, UNITED STATES

## Abstract

**Background:**

Chikungunya virus (CHIKV) is a re-emerging mosquito-borne virus which causes epidemics of fever, severe joint pain and rash. Between 2005 and 2010, the East/Central/South African (ECSA) genotype was responsible for global explosive outbreaks across India, the Indian Ocean and Southeast Asia. From late 2013, Asian genotype CHIKV has caused outbreaks in the Americas. The characteristics of cross-antibody efficacy and epitopes are poorly understood.

**Methodology/Principal Findings:**

We characterized human immune sera collected during two independent outbreaks in Malaysia of the Asian genotype in 2006 and the ECSA genotype in 2008–2010. Neutralizing capacity was analyzed against representative clinical isolates as well as viruses rescued from infectious clones of ECSA and Asian CHIKV. Using whole virus antigen and recombinant E1 and E2 envelope glycoproteins, we further investigated antibody binding sites, epitopes, and antibody titers. Both ECSA and Asian sera demonstrated stronger neutralizing capacity against the ECSA genotype, which corresponded to strong epitope-antibody interaction. ECSA serum targeted conformational epitope sites in the E1-E2 glycoprotein, and E1-E211K, E2-I2T, E2-H5N, E2-G118S and E2-S194G are key amino acids that enhance cross-neutralizing efficacy. As for Asian serum, the antibodies targeting E2 glycoprotein correlated with neutralizing efficacy, and I2T, H5N, G118S and S194G altered and improved the neutralization profile. Rabbit polyclonal antibody against the N-terminal linear neutralizing epitope from the ECSA sequence has reduced binding capacity and neutralization efficacy against Asian CHIKV. These findings imply that the choice of vaccine strain may impact cross-protection against different genotypes.

**Conclusion/Significance:**

Immune serum from humans infected with CHIKV of either ECSA or Asian genotypes showed differences in binding and neutralization characteristics. These findings have implications for the continued outbreaks of co-circulating CHIKV genotypes and effective design of vaccines and diagnostic serological assays.

## Author Summary

Chikungunya virus (CHIKV) has caused large epidemics of fever, rash, and joint pain around the world in recent years. Three different CHIKV genotypes exist. Infection with one genotype is likely to lead to immune protection (or cross-protection) against future infections with a different genotype. However, little is known about the nature of this cross-protection. In this study, we used serum from Malaysian patients infected with CHIKV of either Asian or East/Central/South African (ECSA) genotypes. We compared the ability of the serum antibodies to bind to and neutralize two different viruses, from either Asian or ECSA genotypes. We found that both Asian and ECSA serum were more effective in binding and neutralizing ECSA virus. We identified the key amino acids/epitopes within the E1-E2 surface glycoprotein, and showed that variation of these impacts the efficacy of antiserum in cross-neutralizing different genotypes of CHIKV. We showed how sequence variation of a known linear neutralizing epitope could alter the cross-neutralization efficacy. This study aids understanding of the importance of different circulating genotypes within a country and has implications for the design of vaccines and diagnostic antibody tests.

## Introduction

Chikungunya virus (CHIKV) is a re-emerging, mosquito-borne arbovirus which has caused unprecedented worldwide epidemics in recent years [1]. There are three major CHIKV genotypes circulating: West African, East/ Central/ South African (ECSA) and Asian [2]. After the global outbreaks of ECSA between 2005 and 2010, the Asian genotype has re-emerged to cause large outbreaks in the Americas and the Pacific islands [3, 4]. Malaysia has experienced CHIKV outbreaks due to two different genotypes, Asian and ECSA. The endemic Asian CHIKV strain was responsible for small, geographically-restricted outbreaks in 1998 and 2006 [5–7]. An imported ECSA outbreak was reported in 2006 prior to an explosive nationwide outbreak which affected over 15,000 people across different states in 2008 [8, 9].

CHIKV is an alphavirus from the family *Togaviridae*. A CHIKV virion is 60-70nm in diameter, with a single-stranded positive RNA genome of approximately 11.8 kb in a capsid with a phospholipid envelope carrying glycoproteins E1 and E2. Its genome has 2 open reading frames encoding the non-structural (nsP1-nsP2-nsP3-nsP4) and structural polyproteins (C-E3-E2-6K-E1) [10]. The E1 and E2 glycoproteins form heterodimers which enable interaction with cellular receptors and fusion of the virion envelope with the cell membrane to initiate infection [11], while the capsid protein is required during virus assembly [12]. These proteins are highly immunogenic, and most CHIKV-infected patients develop antibodies targeting the structural proteins (particularly E2) and, to a lesser extent, nsP3 [13, 14]. After the initial induction of type I interferon [15], CHIKV-specific antibodies have been shown as the major effector in immunity to control infection [16]. Among other immune factors, T cells may play a secondary role in suppressing infection [17], although others have found that CD4+ T cells are more important in orchestrating joint inflammation [18].

Currently, treatment for CHIKV is supportive and no licensed vaccine or antiviral are available. Phase I clinical trials have demonstrated the safety and efficacy of vaccination with virus-like particles using structural proteins derived from the West African genotype [19], and a recombinant measles virus-based CHIKV vaccine derived from the ECSA genotype [20]. Cross-reactivity can be achieved against heterogenous genotypes, by which CHIKV seropositive individuals infected with either ECSA or Asian CHIKV have cross-protection against both CHIKV genotypes [9]. However, the cross-neutralizing efficacy of CHIKV-specific antibodies against Asian and ECSA genotypes, which are both currently circulating in Malaysia, Brazil [21] and the Asian region [22], is poorly understood. A distinct antigenic relationship has been established between West African and ECSA genotypes, in which mice and hamsters immunized with the ECSA genotype had 4- to 8-fold differences in neutralizing capacity when tested against a West African strain [2]. In a Singaporean cohort, CHIKV-immune sera exhibited differential antibody binding and neutralizing capacity against isolates with a naturally occurring K252Q amino acid change in the E2 glycoprotein [14]. Given the ability of CHIKV to rapidly spread across different parts of the world with displacement of one genotype with another, the understanding of cross-neutralizing antibody and antigenic variation of different genotypes will have implications for both continued outbreaks and vaccine development.

In this study, we analyzed the neutralizing capacity of CHIKV ECSA and Asian immune sera against representative clinical isolates and rescued viruses of ECSA and Asian CHIKV. We demonstrated that both sets of serum panels have stronger neutralizing capacity against the ECSA isolate, which corresponded to strong epitope-antibody interaction. E1-E211K enhances the neutralization activity of ECSA serum, while E2-I2T, H5N, G118S and S194G within linear epitopes improve the neutralization activity of both sets of sera panels. Rabbit polyclonal antibody targeting a known linear neutralizing epitope (LP1) from ECSA virus could only neutralize homotypic virus, but not heterotypic Asian virus due to sequence variation. These findings indicate the antigenic variation of ECSA or Asian CHIKV genotypes in naturally-acquired infection alters the spectrum of cross-genotype protective antibody immunity.

## Materials and Methods

### CHIKV immune serum panels

This study included 63 human samples from two independent outbreaks in Malaysia. The Asian serum panel comprised 40 samples collected from patients 11–14 months after an Asian CHIKV outbreak in Bagan Panchor in 2006 [7]. The ECSA serum panel consisted of 23 samples from patients infected by ECSA strains in 2008–2010, collected 1–6 months after onset of symptoms, who were seen at the University Malaya Medical Centre in Kuala Lumpur [9]. Healthy controls (*n* = 15) with no past infection of CHIKV served as negative controls. Serum neutralization assay was performed on all the sera. To determine the neutralizing activity due to IgG, heat-inactivated sera were treated for 1 hour with dithiothreitol (DTT) (Life Technologies) at a final concentration of 5mM at 37°C.

### Ethics statement

This study was approved by the Medical Ethics Committee of the University Malaya Medical Centre (reference no. 800.70). Our institution does not require informed consent for retrospective studies of archived and anonymized samples.

### Cells and viruses

Baby hamster kidney (BHK-21) cells (ATCC no. CCL-10) were maintained in Glasgow minimum essential medium (GMEM) (Life Technologies) supplemented with 5% heat-inactivated fetal bovine serum (Flowlab), 10% tryptose phosphate broth, 20mM HEPES, 5mM L-glutamine, 100 U/ml penicillin and 100μg/ml streptomycin. Infected cells were maintained in GMEM containing 2% FBS. The clinical isolates used, which have been previously characterized [23], were MY/06/37348, an Asian genotype strain isolated from a patient in Bagan Panchor in 2006 (accession number FN295483), and MY/08/065, an ECSA virus isolated from a patient in Kuala Lumpur in 2008 (accession number FN295485). Both isolates had been passaged two times in Vero cells (ATCC no. CCL-81) before propagated in BHK-21 cells. Virus passage (P3) of clinical isolates was used for subsequent work.

To study the neutralizing epitopes, viruses rescued from two different infectious clones, derived from ECSA and Asian genotypes of CHIKV, were included. The plasmid vectors capable of producing infectious viruses were constructed under the control of the human cytomegalovirus immediate-early promoter. The CHIKV infectious clone derived from the ECSA genotype was based on LR2006-OPY1, isolated in Reunion Island in 2006,and has been described previously [24]. The full-length infectious cDNA (icDNA) clone from the Asian genotype was engineered by gene synthesis and assembled by the restriction enzymes approach based on the consensus sequence for strain 3462, isolated in Yap State in 2013 (accession no. KJ451623); however, the protein coding regions in the non-structural and structural proteins were changed to be identical to isolate CNR20235 from the Caribbean outbreak, which was isolated in Saint Martin Island in 2013 (http://www.european-virus-archive.com/article147.html). Both molecular clones have ZsGreen gene incorporated as reporter and duplication of the subgenomic promoter. The ECSA molecular clone was named “ICRES1”, while the Asian molecular clone was designated as “CAR”.

For construction of the chimeric viruses, the ectodomain regions of envelope glycoprotein genes E1 (amino acids 1–381) and E2 (amino acids 1–341) in the ICRES1 backbone were replaced with those of Semliki Forest virus (SFV) E1 (amino acids 1–381) and E2 (amino acids 1–340) from icDNA SFV6 [25] using NEBuilder HiFi DNA Assembly Master Mix (NEB). In order to study the effects of point mutations on the neutralizing epitopes, conventional PCR-based site-directed mutagenesis was performed on the CAR construct using Q5 High-Fidelity DNA polymerase (NEB) with designed primers (S1 Table). The sequences of all the constructs were verified by control restrictions and sequence analysis. Primers and sequences for infectious clone constructions are available upon request.

The viruses were rescued from icDNA by electroporation. Stocks of rescued viruses (P0) were harvested and titrated by plaque assay on BHK-21 cells. To obtain P1 stocks, confluent BHK-21 cells grown in T75-cm^2^ flasks were infected with P0 stocks at a multiplicity of infection (MOI) of 1 plaque forming unit/cell and maintained in 2% FBS GMEM. P1 stocks were harvested after 24 or 48 hours, titrated and used for the neutralization assay. Infectious center assay was performed on all the viruses rescued from icDNA. Details on virus rescue and related protocols are shown in S1 Text and S2 Table.

### Serum neutralization assay

Seroneutralization was performed with a previously described immunofluorescence-based cell infection assay in BHK-21 cells [26, 27], with minor modifications. The DTT-treated sera underwent 2-fold serial dilutions (1:100 to 1:6400) in 1X Dulbecco’s PBS prior to mixing with CHIKV pre-diluted with 2% FBS GMEM. Cells were infected with clinical isolates at an MOI of 10. The virus-antibody mixture was incubated for 2 hours at 37°C before inoculation into 10^4^ cells in 96-well CellCarrier-96 optic black plates (Perkin Elmer), and further incubated for 1.5 hours at 37°C. The inocula were decanted and 2% FBS GMEM was added to the plates. The plates were fixed with 4% paraformaldehyde after 6 hours of incubation at 37°C, permeabilized with 0.25% Triton X-100 for 10 minutes, and immunostained using anti-CHIKV E2 monoclonal antibody B-D2(C4) [28] at 1μg/ml followed by rabbit anti-mouse IgG-FITC (Thermo Scientific) at 1:100 dilution. Cell nuclei were counter-stained with DAPI. Fluorescence intensity was analyzed with a Cellomics High Content Screening (HCS) ArrayScan VTI (Thermo Fisher) over 9 different fields at 5X magnification. Percentage of infectivity was calculated according to the following equation: % infectivity = (mean average fluorescence intensity from serum sample/mean average fluorescence intensity from virus control) × 100. The neutralizing titer (NT_50_) was expressed as the serum dilution that reduced infectivity by 50% using non-linear regression fitting in GraphPad Prism 5 (GraphPad Software).

For seroneutralization using rescued viruses, diluted sera were mixed with viruses pre-diluted with 2% FBS GMEM (with infection performed at an MOI of 50), followed by the steps described above. The plates were fixed after 7 hours of incubation at 37°C. The plates were only counter-stained with DAPI prior to acquisition of ZsGreen fluorescence.

To investigate the cross-reactivity of CHIKV sera against another alphavirus, SFV was rescued from icDNA SFV6 as previously described [25]. Diluted sera (1:25 and 1:100 dilutions) were mixed with SFV pre-diluted with 2% FBS GMEM (with infection performed at an MOI of 10), followed by the steps described above. The plates were fixed after 6 hours of incubation at 37°C, and stained with mouse anti-alphavirus monoclonal antibody (Santa Cruz) at 1:100 dilution.

To investigate the effect of sequence variation of neutralizing epitopes in ECSA and Asian genotypes, polyclonal rabbit anti-LP1 (STKDNFNVYKATRPY), anti-LP1A (SIKDHFNVYKATRPY) and anti-LP47 (NHKKWQYNSPLVPRN) were produced commercially (GenScript). LP1 is similar to E2EP3, an immunogenic peptide (from an ECSA virus) previously reported to elicit neutralizing antibodies [26]. LP1A is the corresponding variant peptide with Asian genotype sequences. The LP47 peptide sequence is conserved in both genotypes. Seroneutralization was performed with purified antibody at 25μg/ml against the rescued viruses.

### Whole virus antigens and recombinant proteins

For indirect IgG ELISA (antibody end-point assay) and Western blot, the antigen was partially purified virus prepared by sucrose-cushion ultra-centrifugation, treated with 1% Triton X-100 in TE buffer, clarified by centrifugation, and stored in 50% glycerol at -20°C.

For production of native recombinant proteins of E1 (rE1, from amino acids 1–412) and E2 (rE2, from amino acids 1–362), viral RNA was extracted from clinical isolates (Asian MY/06/37348 and ECSA MY/08/065). cDNA was synthesized using reverse-transcription, and the genes were amplified using high fidelity Platinum Taq (Invitrogen) with designed primers (S3 Table). The transmembrane regions and cytoplasmic tails of the glycoproteins were not included in the expression cassette, to ensure solubility of the recombinant proteins. The amplicons were ligated into a pIEX-5 vector (Novagen) directionally at *BamH1* and *Not1* restriction sites. Each plasmid construct together with a pIE1-neo vector were co-transfected into Sf9 cells (Novagen) using Cellfectin II reagent (Invitrogen) [29]. Stable clones expressing rE1 and rE2 were generated under selection with G418 sulfate at 1000μg/ml. The proteins secreted from stable clones were purified under native conditions with activated Profinity IMAC resins (Bio-Rad) or HisTrap FF (GE). The eluates were concentrated with an Amicon centrifugal unit and the buffer was exchanged with sodium phosphate buffer (50mM NaH_2_PO_4_, 300mM NaCl, pH 8.0). The proteins were stored at -20°C in 50% glycerol, except for the proteins used in the competitive protein blocking assay, which were filter-sterilized and kept at 4°C. Fusion sequences expressing for rE2 and rE1 was generated by overlapping PCR; recombinant proteins encoded by obtained sequence were linked via linker with sequence GGGS-His (8X)-GGGG (S1 Text). The fusion glycoprotein constructs were transfected into TriExSf9 cells (Novagen) by TransIT-Insect transfection reagent (Mirus Bio).

### Western blot

The proteins were resolved with 12% SDS-PAGE under reducing and non-reducing conditions and electro-transferred onto a nitrocellulose membrane (GE). The membrane was blocked with 10% skimmed milk in 0.05% PBS-Tween 20 (PBST). The immunoreactivity of recombinant proteins was evaluated with pools of CHIKV immune sera applied at indicated dilutions in the blocking buffer. The bound antigen-antibody complex was detected by anti-human IgG-HRP (DakoCytomation) at 1:5000 dilution in 1% bovine serum albumin (BSA)-0.05% PBST. The membrane was visualized by chemiluminescence (Bio-Rad) and images were acquired with a BioSpectrum AC imaging system (UVP). Mouse anti-His tag antibody (Merck Millipore) was included as a loading control. Mouse anti-E2 monoclonal antibody (clone: B-D2(C4); EIEVHMPPDT) [28] was also included as a control.

### Enzyme-linked immunosorbent assay (ELISA)

All incubation steps were performed at 37°C for 1 hour, using 1% BSA-0.05% PBST as diluent for serum and antibodies. The plates were washed 4 times with 0.05% PBST after each incubation step. To determine the relative level of anti-E2 antibodies, the plates were coated with 250 ng of virus antigen or 100 ng of rE2 in 0.05M carbonate-bicarbonate buffer (pH 9.6). The antigens were normalized with monoclonal antibody B-D2(C4) to determine the relative level of anti-E2 antibodies. The plate was blocked with 3% BSA in 0.05% PBST. The sera were tested at 2-fold serial dilutions from 1:512 to 1:1,048,000 or 1:640 to 1:655,000. The IgG end-point titer was determined as the reciprocal of the highest dilution that produced an optical density (OD) reading of three times greater than that of the negative control. Anti-human IgG-HRP at 1:5000 dilution was added to detect the bound antibodies. TMB substrate (KPL) was added to each well and the plates were incubated at room temperature for 5 min. The reaction was terminated by adding 1M phosphoric acid. The absorbance was measured at 450nm with 630nm as the reference wavelength using an automated ELISA reader (Biotek Instruments). The cut-off value was established as the OD obtained from healthy controls sera plus three standard deviations (SD). The relative level of anti-rE2 antibodies was calculated with the following formula: (end point titer for rE2/end point titer for whole virus antigen) × 100.

### Competitive protein/peptide blocking assay

Soluble recombinant CHIKV proteins (15μg) were mixed with heat-inactivated immune sera diluted at 1:200, and incubated for 1 hour at 37°C. CHIKV (MY/08/065) in amounts corresponding to an MOI of 10 was mixed with the samples, which were incubated for a further 2 hours at 37°C.

Synthetic peptides were obtained from GenScript (LP1, STKDNFNVYKATRPY; LP24, TDSRKISHSCTHPFH; LP38, GNVKITVNGQTVRYK); 60μg of each peptide was mixed with immune sera diluted with 1X DPBS at 1:100 and incubated for 1.5 hours at 37°C. All the synthetic peptides for the blocking assay have a purity grade greater than 95% and are soluble in high-grade water. ICRES1 (sucrose-cushion purified virus in TE buffer pre-diluted using 2% FBS GMEM) at an amount corresponding to an MOI of 1 was mixed with the samples, which were incubated for a further 2 hours at 37°C prior to infection of BHK-21 cells. The plate was replenished with plaque medium (2% FBS GMEM containing 0.8% of carboxymethylcellulose), fixed with 4% paraformaldehyde after 15 hours of incubation, and this was followed by ZsGreen fluorescence acquisition. Infectivity corresponded to the fluorescence intensity acquired with a Cellomics HCS reader. The effect on infectivity of antibodies in the presence and absence of blocking peptides was compared.

### Peptide-based ELISA and epitopes analysis

Biotinylated synthetic peptides covering the E2 glycoprotein sequence from amino acids 1–362 from a previous study [28] were used to screen CHIKV immune sera for binding to linear epitopes. The length of each peptide is 15-mer with a 10-mer overlap based on the CHIKV MY/08/065 sequence (accession no. FN295485; S4 Table). Similar steps were performed as described above except that the plates were washed 6 times after incubation with human sera and secondary antibody. The plates were coated with 20μg/ml streptavidin (NEB) and blocked with 5% BSA-PBST. The dissolved peptides in dimethyl sulphoxide were further diluted to a working concentration of approximately 150μg/ml in 1% BSA-PBST. CHIKV immune sera and healthy control sera were diluted at 1:1000, and screened against peptides in duplicate. The peptides with the highest OD reading from 2 adjacent overlapping synthetic peptides were considered as identified B-cell epitopes. Computational analysis and epitope localization were performed on structural data retrieved from Protein Data Bank (PDB, ID 3J2W) with UCSF CHIMERA software [30]. As the LP1 sequence is unresolved in structural data, the structure of the E2 glycoprotein was predicted using the online I-TASSER server [31, 32]. The electrostatic potential of the E2 structure (amino acid 1–362) was evaluated with PDB2PQR and APBS [33–35].

### Statistical analysis

Data are presented as means ± SD or means ± standard error of the mean (SEM). Differences between groups and controls were analyzed using appropriate statistical tests. A *P*-value of <0.05 was considered significant. Statistical analyses were performed with GraphPad Prism 5.

## Results

### Both ECSA and Asian sera have greater neutralizing capacity and binding with the ECSA genotype

**Fig 1:**
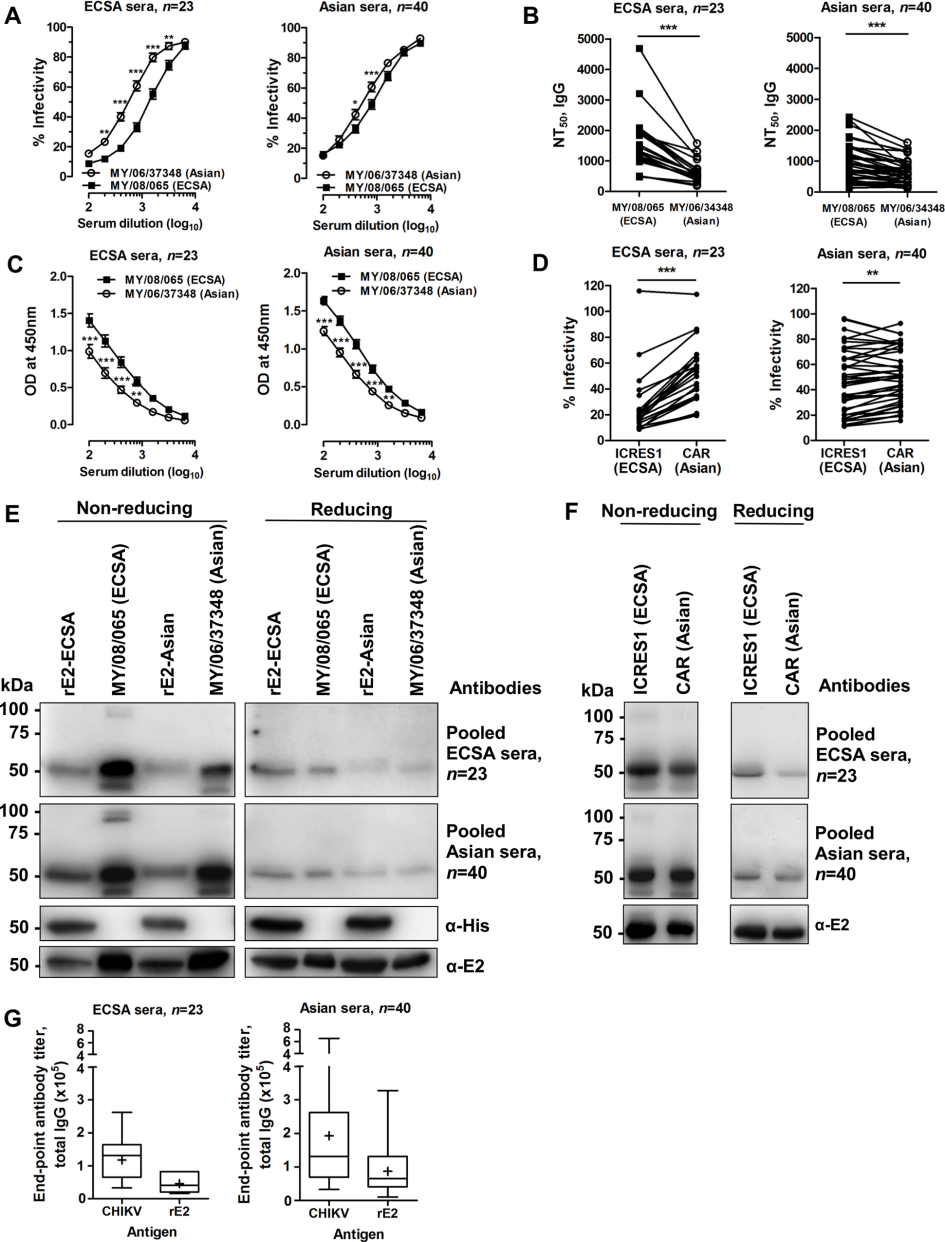
Differential neutralization capacity and antibody binding properties of immune sera against ECSA and Asian CHIKV. (A) Sera collected from ECSA and Asian CHIKV outbreaks have differential neutralizing capacity against MY/08/065 (ECSA) and MY/06/37348 (Asian) isolates of CHIKV. Results are expressed as a percentage of virus control. *P<0.05, **P<0.01, ***P<0.001, two-way ANOVA with the Bonferroni multiple comparisons test. Data are presented as means ± SEM from 23 (ECSA) and 40 (Asian) individual serum samples. (B) Neutralization titers (NT_50_) of DTT-treated sera were determined by non-linear regression fitting. ***P<0.001, Wilcoxon matched-pairs signed rank test. (C) ECSA and Asian sera were cross-screened against both CHIKV isolates (10^5^ pfu, treated with 1% Triton X-100) in ELISA at different serum dilutions. Data are presented as means ± SEM from 23 (ECSA) and 40 (Asian) individual serum samples. **P<0.01, ***P<0.001, two-way ANOVA with the Bonferroni multiple comparisons test. (D) Seroneutralization was performed against different strains of CHIKV, ICRES1 (ECSA) and CAR (Asian), which were rescued from icDNA CHIKV, at a serum dilution of 1:800. **P<0.01, ***P<0.0001, Wilcoxon matched-pairs signed rank test. (E) Immunoblotting was performed under non-reducing and reducing conditions against rE2 and CHIKV from MY/08/065 and MY/06/37348. Mouse anti-His was used as a control and pooled sera were diluted at 1:1000. (F) Immunoblotting was performed under non-reducing and reducing conditions against ICRES1 and CAR. Mouse anti-E2 was used as a control and pooled sera were diluted at 1:1000. (G) Antibody titers of CHIKV immune sera (IgG) were quantified by end-point titer ELISA using whole virus antigen and recombinant E2 (rE2) derived from MY/08/065. Middle line, median; plus sign, mean; upper and lower boundaries of the box, inter-quartile range; whiskers, range of values.

We employed a sensitive seroneutralization assay to compare the neutralizing capacity of the different sera panels against MY/08/065 (ECSA) and MY/06/37348 (Asian) (S1 and S2 Figs). The heat-inactivated intact sera and DTT-treated sera had similar neutralizing capacity against MY/08/065 for both sera panels (S3 Fig). ECSA sera demonstrated strong neutralizing capacity against homotypic CHIKV compared to heterotypic CHIKV (Fig 1A), with a NT_50_ against MY/08/065 that was a median 2.67 (range, 1.40–4.61) times greater than the NT_50_ against MY/06/37348 (Fig 1B). Unexpectedly, Asian sera demonstrated better neutralizing capacity against heterotypic ECSA CHIKV compared to homotypic CHIKV (Fig 1A), with a NT_50_ against MY/08/065 of a median 1.44 (range, 0.70–3.19) times greater than the NT_50_ against MY/06/37348 (Fig 1B). The greater neutralizing capacity corresponded to stronger antibody binding to MY/08/065 compared to MY/06/37348 by quantitative ELISA (Fig 1C). Seroneutralization was performed against rescued virus from icDNA of ECSA and Asian genotypes. Both ECSA and Asian sera demonstrated better neutralizing capacity against ICRES1 (ECSA) compared to CAR (Asian) (Fig 1D). Immunoblotting showed stronger reactivity of serum with the whole viral antigen (with a band of about 50kDa, consistent with E1 or E2, a known immunodominant antigen in alphaviruses) and recombinant E2 glycoprotein of similar size derived from ECSA, compared to the Asian genotype. Under non-reducing conditions, ECSA sera had stronger antibody binding to its homotypic CHIKV isolate MY/08/065, ICRES1 and recombinant E2 glycoprotein (rE2) (Fig 1E and 1F). Asian sera bound similarly to both genotypes of viruses (clinical isolates and rescued viruses), and more strongly to rE2 glycoprotein of MY/08/065 (Fig 1E and 1F). Under reducing conditions, both sets of sera retained stronger binding to ECSA CHIKV and rE2 of ECSA CHIKV. Both sets of sera had a similar proportion of total antibodies binding to rE2 (median 50%, range 20–63% for ECSA serum; median 50%, range 16–63% for Asian serum) (Fig 1G), and these percentages suggest that antibodies also target sites other than E2. Taken together, CHIKV serum shows strong neutralizing capacity and binding to CHIKV, particularly of the ECSA genotype, and the epitopes may be presented as part of the conformational E1-E2 glycoprotein and/or as linear determinants in the E2 glycoprotein.

### ECSA and Asian sera target epitopes on the E1-E2 glycoprotein

**Fig 2:**
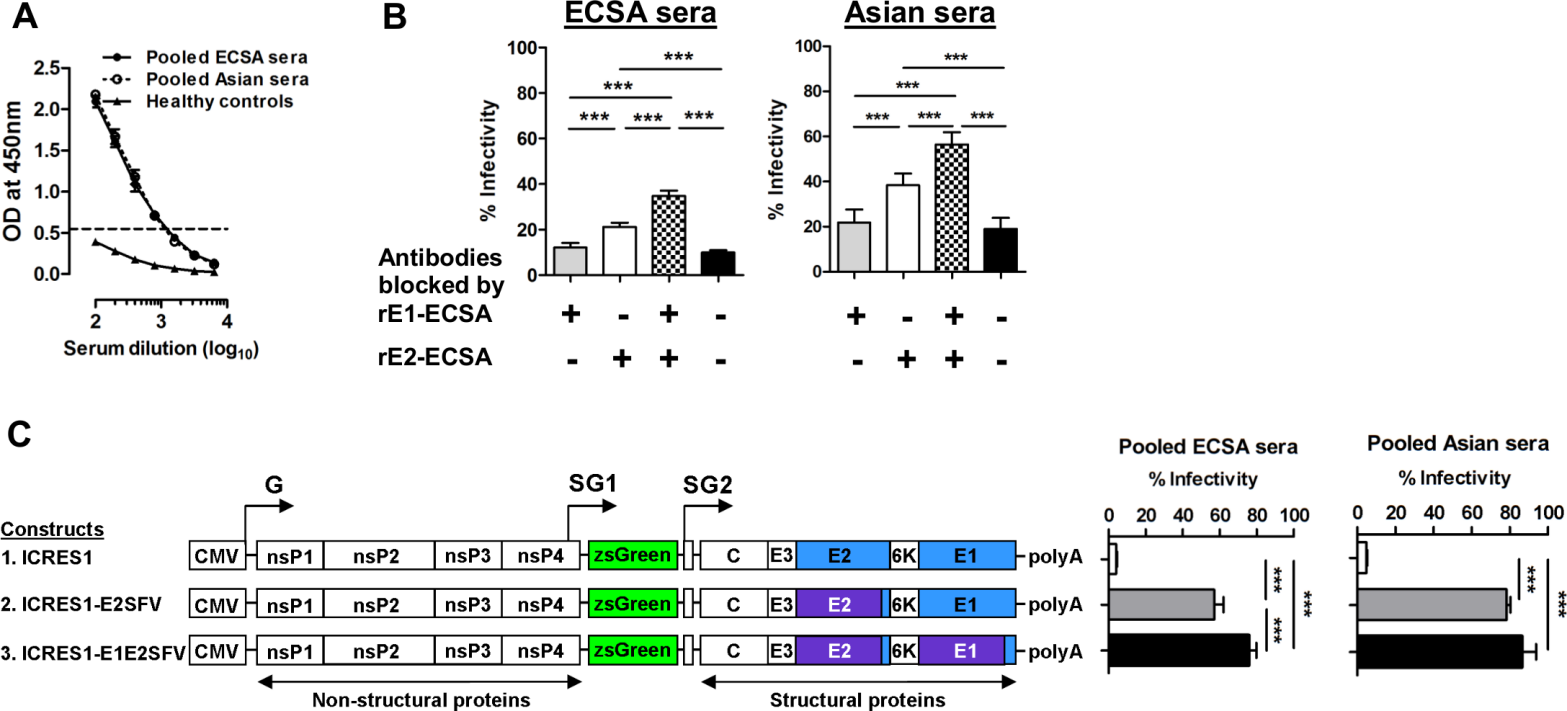
Neutralizing antibodies of immune sera interact with the epitopes on E2 and E1-E2 glycoproteins. (A) CHIKV antibody titer against recombinant E1 glycoprotein (100 ng) was determined in ELISA. The ELISA was performed at different serum dilutions using pooled sera. The dotted line represents the cut-off value (mean + 3SD) derived from healthy controls. (B) Competitive blocking assay was performed at 1:200 dilution in triplicate using 7 pools of ECSA and Asian sera, with similar neutralizing titers in each pool. Data are expressed as percentages of infectivity of an infection control, and are presented as means ± SEM. ***P< 0.001, repeated measures ANOVA with the Bonferroni multiple comparison test. (C) Schematic diagram showing the construction of chimera viruses with replacement of E2 or E1/E2 from SFV into the CHIKV ICRES1 backbone. Seroneutralization was performed against the chimera constructs and the percentage of infectivity was compared to that obtained with ICRES1. Data are represented as means ± SD from 4 independent experiments at a serum dilution of 1:100 (pooled sera). ***P<0.01, Kruskal-Wallis test. G, genomic promoter; SG, subgenomic promoter.

To determine if CHIKV immune serum targets E1, recombinant E1 glycoprotein (rE1) was probed in ELISA with serially diluted pooled sera, and signal was detected at low serum dilutions from 1: 100 to 1:800 (Fig 2A). A competitive protein blocking assay was performed, and blocking of ECSA and Asian sera with native rE1 alone did not significantly alter the neutralizing capacity (Fig 2B). However, when the sera was blocked by a mixture of rE1 and rE2, significant increases of infectivity were observed in both panels of sera compared to unblocked sera or sera blocked by either rE1 or rE2 alone. We then hypothesized that antibodies may target conformational epitopes on E1 and E2 glycoproteins together. To test this hypothesis, we constructed 2 chimeras which swapped the ecto-domain regions of the E2 and E1-E2 glycoproteins with those of Semliki Forest virus (SFV). Both sets of sera demonstrated a low degree of cross-neutralization against SFV, another alphavirus which is a member of the same antigenic complex as CHIKV (S4 Fig). At 1:100 serum dilution, loss of neutralizing effect for both sets of sera was observed when CHIKV E2 was replaced with SFV E2. Furthermore, in ECSA serum, loss of neutralization activity was much higher against the chimera with E1-E2 from SFV compared to the chimera with SFV E2 alone (Fig 2C). This provides further evidence that neutralizing antibodies are not solely targeting E2, but are also targeting epitopes spanning E1-E2 glycoproteins. Alternatively, E1 may affect the conformation of E2 and alter its epitopes.

### The E1-E211K amino acid change enhances neutralization activity of ECSA serum

**Fig 3:**
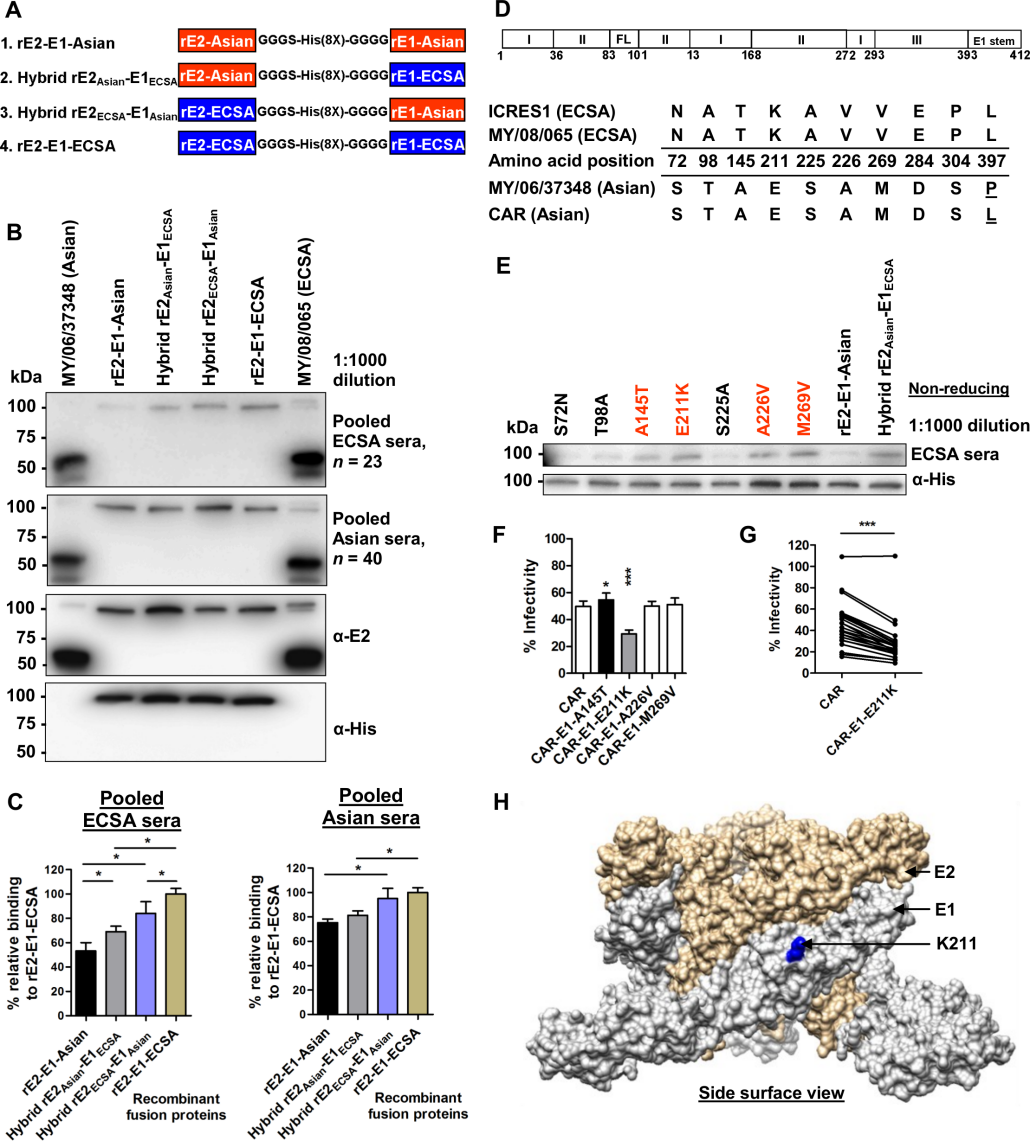
The E1-E211K amino acid change enhances neutralization activity of ECSA serum. (A) Schematic diagram showing the generation of fusion recombinant E2 (amino acids 1–362) and recombinant E1 (amino acids 1–412) with a 16 residue linker which has glycine/ serine spacers and octa-histidine sequence. The rE2 and rE1 in each fusion protein are from either MY/08/065 (ECSA) or MY/06/37348 (Asian) virus isolates. (B) Immunoblotting was performed under non-reducing condition. Mouse anti-E2 and mouse anti-His monoclonal antibodies were used as controls. (C) The relative binding capacity of ECSA and Asian sera (1:1000 dilution) with the fusion E2-E1 proteins were determined in ELISA as (OD samples/mean OD samples tested with rE2-E1-ECSA) × 100. Data are presented as means ± SD (*n* = 4). *P<0.05, Mann-Whitney U test. (D) Schematic representation of the E1 glycoprotein with the numbers indicating the amino acid positions of the glycoprotein and its domain proteins. The amino acid differences between E1 glycoprotein of ECSA (MY/08/065, ICRES1) and Asian (MY/06/37348, CAR) were tabulated and mapped (from amino acids 1–412). Amino acid differences within a genotype are underlined. FL, fusion loop; C. tail, cytoplasmic tail. (E) Immunoblotting was performed against fusion E2-E1 glycoprotein under non-reducing conditions, with each named amino acid change from the Asian to the ECSA sequence introduced independently. Mouse anti-His antibody was used as a control. (F) Seroneutralization was performed against different constructs carrying indicated mutations in the CAR-2SG-ZsGreen backbone, which were rescued from the corresponding icDNA clones of CHIKV. Data are represented as means ± SD from 4 independent experiments at a serum dilution of 1:800 (pooled sera). *P<0.05, ***P<0.001, Mann-Whitney U test, relative to CAR. (G) Seroneutralization was performed against the CAR-E1-E211K rescued virus at a serum dilution of 1:800 with 23 individual serum samples. ***P<0.0001, Wilcoxon matched-pairs signed rank test. (H) The amino acid position of K211 which affects neutralization activity is localized on the structural E1-E2 heterodimer complex (based on PDB 3J2W).

To further determine the importance of conformational epitopes resulting from interactions between E1 and E2, four sets of fusion E1-E2 glycoproteins were constructed. Each hybrid fusion protein contained E1 and E2 sequences from either MY/06/37348 (ECSA) or MY/08/065 (Asian), transiently expressed as secreted native recombinant proteins in insect cells (Fig 3A). The antibody binding capacity of ECSA sera against fusion E1-E2 proteins significantly increased when either the E1 or E2 sequence was changed from that of MY/06/37348 to that of MY/08/065, as shown in immunoblotting (Fig 3B) and quantitative ELISA (Fig 3C). Asian sera had almost equal antibody binding capacity for the 4 fusion glycoproteins, suggesting that Asian serum was not sensitive to sequence changes in E1-E2 glycoproteins. This data shows that the greater binding and neutralization of the ECSA isolate MY/08/065 by ECSA sera (Fig 1A, 1C and 1D) is due to critical conformational epitopes on the E1-E2 heterodimer, which are sequence-dependent.

Between ECSA (MY/08/065 and ICRES1) and Asian (MY/06/37348 and CAR) genotypes of CHIKV in this study, there are 10 amino acids differences in E1 (Fig 3D). Using the fusion rE2-E1-Asian construct as a template, site-directed mutagenesis was performed independently to replace each amino acid of Asian origin with the corresponding ECSA residue, and the proteins were expressed in insect cells. The antibody binding significantly increased with the amino acid changes at A145T, E211K, A226V and M269V, in comparison to hybrid rE2_Asian_-E1_ECSA_ recombinant proteins (Fig 3E). Recombinant virus carrying E1-211K demonstrated a large increase in neutralizing capacity compared to the parental virus clone (CAR), while the E1-145T change caused a slight decrease in neutralizing capacity (Fig 3F and 3G). The critical 211K amino acid was localized at the surface of E1-E2 heterodimers (Fig 3H).

### I2T, H5N, G118S and S194G substitutions within linear neutralizing epitopes of E2 glycoprotein enhance the neutralization activity of ECSA and Asian sera

**Fig 4:**
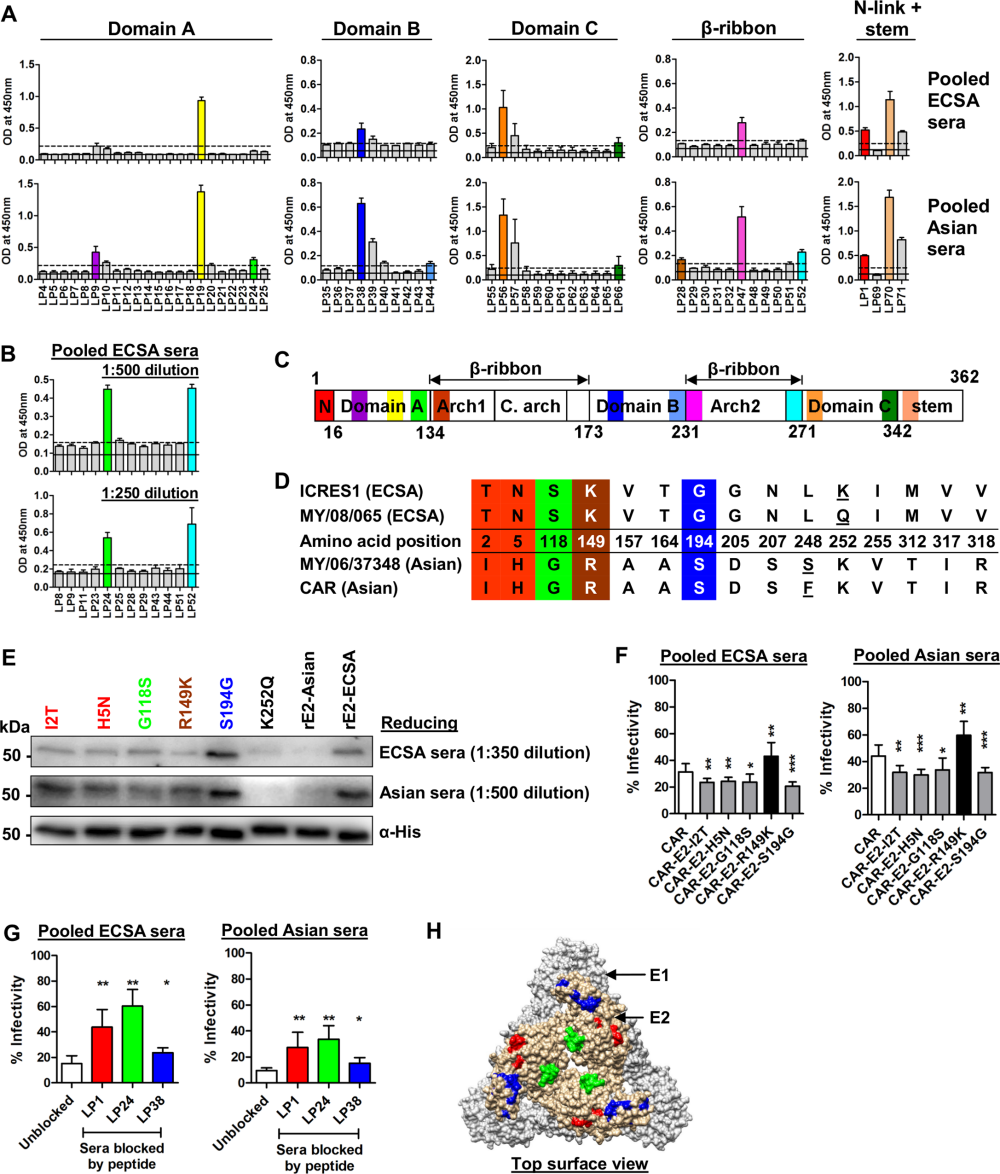
E2-I2T, H5N, G118S and S194G substitutions within linear neutralizing epitopes enhance the neutralization activity of ECSA and Asian sera. (A) Overlapping synthetic peptides covering the E2 glycoprotein of MY/08/065 and its domains from amino acids 1–362 were screened with CHIKV immune sera at 1:1000 dilution. The black solid line represents the mean OD value of healthy controls and the dotted line represents the cut-off value (mean+3SD). The average results from 2 independent experiments are presented. In the event of two adjacent positively-mapping peptides, the peptide with the highest OD reading was taken. Key positive mapping peptides are color-coded. (B) Selected synthetic peptides were re-screened with pooled ECSA sera at lower dilutions of 1:500 and 1:250, in tetraplicate. The black solid line represents the mean OD value of healthy controls and the dotted line represents the cut-off value (mean+3SD). Key positive mapping peptides are color-coded. (C) Schematic diagram of the E2 protein showing the positions of the color-coded mapped epitopes. The numbers refer to the amino acid positions demarcating the E2 domains. N, N-link; C.arch, central arch. (D) Schematic representation of the E2 glycoprotein with the numbers indicating the amino acid positions of the glycoprotein and its domain proteins. The amino acid differences between E2 glycoproteins of ECSA (MY/08/065, ICRES1) and Asian (MY/06/37348, CAR) strains were tabulated and mapped (from amino acids 1–362). Amino acid differences within a genotype are underlined. Amino acid changes which fall within the identified linear epitopes are color-coded. (E) Immunoblotting was performed against recombinant E2 glycoproteins under reducing conditions, with each named amino acid change from the Asian to the ECSA sequence introduced independently. Mouse anti-His antibody was used as a control. (F) Seroneutralization was performed against different constructs with the CAR-2SG-ZsGreen backbone, which were rescued from the corresponding CHIKV icDNAs. Data are represented as means ± SD from 4 independent experiments at a serum dilution of 1:800 (pooled sera). *P<0.05, **P<0.01, ***P<0.001, Mann-Whitney U test. (G) Competitive peptide blocking assay was performed at 1:100 dilution with either pooled ECSA or Asian sera against ICRES1 at an MOI of 1. Data are expressed as percentages of infectivity of an infection control, and are presented as means ± SD from 2 independent experiments. *P<0.05, **P< 0.01, Mann-Whitney U test, relative to unblocked condition. (H) The color-coded mapped neutralizing epitopes are localized on the structural E1-E2 heterodimer complex (based on PDB 3J2W). The epitope sequence of LP1 is only partially localized as the 3D structure is not fully resolved.

To study the linear epitopes in the immunodominant E2 glycoprotein (based on strain MY/08/065, of the ECSA genotype), overlapping synthetic peptides covering amino acids 1–362 were mapped by peptide-ELISA using the ECSA and Asian sera (Fig 4A, 4B and 4C). Both ECSA and Asian sera mapped to the same 9 peptides, and the Asian sera mapped to an additional 3 peptides (Table 1). Between the strains of ECSA (MY/08/065, ICRES1) and Asian (MY/06/37348, CAR) genotypes of CHIKV used in this study, there are 15 amino acid differences in E2 (from amino acids 1–362), of which 4 amino acid differences fall within the identified linear epitopes (Fig 4D). Using the rE2-Asian construct as a backbone, site-directed mutagenesis was performed to replace each amino acid of Asian origin with an ECSA residue, and the proteins were expressed in insect cells. The antibody binding significantly increased with I2T, H5N, G118S, R149K and S194G substitutions in comparison to the original rE2-Asian recombinant protein (Fig 4E and S5 Fig). Recombinant viruses carrying either E2-2T, 5N, 118S or 194G demonstrated increases in neutralizing capacity compared to the parental virus clone (CAR), while the E2-R149K change caused a decrease in neutralizing capacity (Fig 4F). Competitive peptide blocking assay indicated that the anti-CHIKV antibodies interact with the LP1, LP24 and LP38 peptides that cover amino acid sites 2, 5, 118 and 194 on E2 (Fig 4G). These 4 neutralizing linear epitopes are localized on the surface of the E1-E2 heterodimer complex (Fig 4H).

**Table 1:** Sequences of identified B cell epitopes on the E2 glycoprotein of MY/08/065.

Domain binding site	B cell epitope sequence ^a^	Amino acid positions ^b^	Peptide annotation
N-link	STKDNFNVYKATRPY	1–15	LP1
A	ATDGTLKIQVSLQIG	41–55	LP9
	CTITGTMGHFILARC	91–105	LP19
	TDSRKISHSCTHPFH	116–130	LP24
β-ribbon (Arch1)	IGREKFHSRPQHGKE	136–150	LP28
B	GNVKITVNGQTVRYK	186–200	LP38
	VINNCKVDQCHAAVT	216–230	LP44
β-ribbon (Arch 2)	NHKKWQYNSPLVPRN	231–245	LP47
	HIPFPLANVTCRVPK	256–270	LP52
C	VTYGKNQVIMLLYPD	276–290	LP56
	LEVTWGNNEPYKYWP	326–340	LP66
Stem	GTAHGHPHEIILYYY	346–360	LP70

^a^ Underlined sequences indicate common epitopes recognised by both ECSA and Asian sera.

^b^ The first amino acid in E2 is numbered as 1.

### Sequence variation of a neutralizing linear epitope influences cross-genotype neutralization

**Fig 5:**
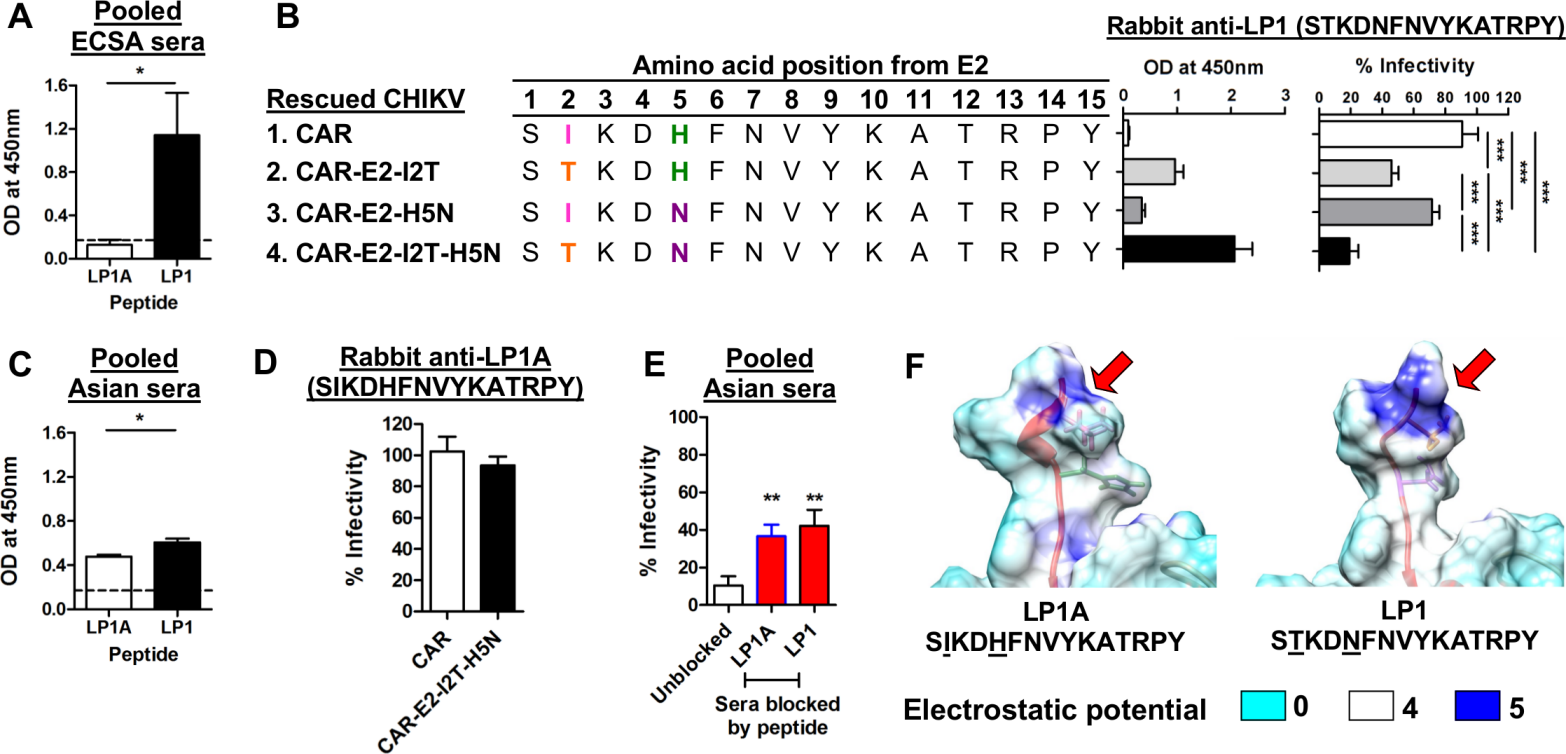
Sequence variation of a linear neutralizing epitope influences the spectrum of cross-neutralization across genotypes. (A) Synthetic peptides LP1A and LP1 were screened with ECSA immune sera at 1:500 dilution in tetraplicate. The dotted line represents the cut-off value (mean + 3SD). *P<0.05, Mann-Whitney U test. (B) Recombinant viruses were pre-treated with 1% Triton X-100, then coated at 5 x 10^5^ pfu per well, and a binding assay was performed with 1μg/ml of antibody. The seroneutralization assay was performed with infection at an MOI of 50 against 25μg/ml of antibody. Data are represented as means ± SD from 3 independent experiments. ***P<0.001, repeated measures ANOVA with the Bonferroni multiple comparisons test. (C) Synthetic peptides LP1A and LP1 were screened with Asian immune sera at 1:500 dilution in tetraplicate. The dotted line represents the cut-off value (mean + 3SD). *P<0.05, Mann-Whitney U test. (D) Seroneutralization of anti-LP1A against CAR and CAR-E2-I2T-H5N at an antibody concentration of 25 μg/ml. Data are represented as means ± SD from 3 independent experiments.(E) Competitive peptide blocking assay was performed at 1:100 dilution with either LP1A or LP1 in pooled Asian sera against ICRES1 at an MOI of 1. Data are expressed as percentages of infectivity of an infection control, and are presented as means ± SD (*n* = 6). **P< 0.01, Mann-Whitney U test, relative to unblocked condition. (F) Structural images illustrating the changes of surface electrostatic potential due to differences in amino acid positions 2 and 5 within LP1A (Asian) and LP1 (ECSA) sequences (red arrows).

As naturally-acquired infection of the Asian genotype of CHIKV leads to higher cross-neutralizing efficacy against ECSA CHIKV, we hypothesized that an epitope-based vaccine derived from the Asian genotype might provide a substantial level of cross-protection against ECSA CHIKV. The peptide LP1 (STKDNFNVYKATRPY) is similar to E2EP3, a peptide derived from ECSA virus which has been found to be highly immunogenic in eliciting neutralizing antibodies in an animal model [26]. We generated a variant, LP1A (SIKDHFNVYKATRPY), derived from the sequence of the Asian virus. Rabbit polyclonal antibodies were commercially prepared against LP1A and LP1. Peptide-ELISA was performed using human ECSA and Asian serum with LP1A and LP1 as antigens. Human ECSA serum bound to LP1 but not LP1A (Fig 5A). Rabbit anti-LP1 antibody showed the lowest binding capacity against CAR (Asian), and demonstrated poor neutralizing activity against the CAR virus harboring the LP1A sequence (infectivity 91±10%, Fig 5B). Anti-LP1 binding capacity and neutralization efficacy was partially restored with the mutations I2T and H5N. The anti-LP1 antibody had maximum binding capacity and neutralizing efficacy against CAR-E2-I2T-H5N (Fig 5B), which has the LP1 sequence; a finding in line with the antibody binding of ECSA immune sera against LP1 peptide (Fig 5A).

Asian serum could recognize LP1A, although binding was marginally higher to LP1 (Fig 5C), which supports the earlier finding that Asian serum has stronger binding against LP1 with I2T and H5N amino acid changes (Fig 4E). Unexpectedly, rabbit anti-LP1A did not demonstrate significant neutralizing activity against CHIKV with either the LP1A or LP1 sequences (Fig 5D). However, a competitive peptide blocking assay indicated that neutralizing antibodies from Asian sera could still recognize and interact with both LP1A and LP1 peptides (Fig 5E). The electrostatic potential of the E2 surface was computed based on the CAR ecto-domain region to study the charge distribution of these epitopes which affect binding affinity [36]. The I2T change leads to higher electrostatic potential, which is associated with improved binding capacity and neutralization efficacy (Fig 5F). LP47, another linear neutralizing epitope in humans, also failed to induce any functional neutralizing antibodies in rabbits.

## Discussion

CHIKV has become a major public health concern worldwide and causes considerable socio-economic burden. Protective adaptive immunity is mainly provided by specific antibodies, particularly those directed against epitopes on the E2 and E1 glycoproteins [37, 38]. Understanding cross-immunity resulting from infections with different genotypes is particularly important and timely. Many Asian countries now have both endemic Asian and epidemic ECSA strains circulating, and the recent widespread outbreaks in the Americas are due to the Asian genotype rather than the previously epidemic ECSA strains, indicating that viruses from both genotypes are capable of global spread.

In this study, we showed differences in cross-genotypic neutralization efficacy of immune sera against ECSA and Asian genotypes of CHIKV. Both ECSA and Asian serum had greater neutralizing capacity against ECSA genotype (MY/08/065 and ICRES1) than Asian genotype (MY/06/37348 and CAR), indicating that neutralizing antibodies regardless of initial infecting genotype preferentially recognized the epitopes presented by the ECSA genotype. The presence of cross-genotype neutralization was clearly shown lasting up to 14 months post-infection. The clinical significance of the differential cross-protective capacity of ECSA and Asian sera remains unclear, as all the immune sera had more than the minimum neutralizing titer (≥10) which appears to correlate with immune protection from symptomatic CHIKV infection in humans [39]. This high degree of cross-neutralization likely contributed to the geographic restriction of CHIKV of different genotypes seen historically, which limited, for example, the spread of ECSA viruses in Asia, at least until CHIKV underwent mutations that facilitated sequential adaptation to the *Aedes albopictus* vector [40, 41].

Apart from the stronger antigenicity of epitopes of the ECSA genotype, we also showed that neutralizing capacity was also affected by the target and the amount of neutralizing antibodies. Both ECSA and Asian sera contain high levels of neutralizing antibodies to numerous linear epitopes on the E2 glycoprotein as well as conformational epitopes on the E1-E2 heterodimer complex. This supports recent findings that most of the reported CHIKV neutralizing monoclonal antibodies target conformational epitopes on the exposed, topmost outer surfaces of the E2/E1 spike, particularly in domain A and domain B [42–45]. Our findings also suggest that subunit vaccine candidates derived from E1 or E2 glycoproteins alone [46–48] may be insufficient to provide full protection against all genotypes, and that virus-like particle vaccines which present epitopes on E2/E1 in their native configuration may preferentially induce the most highly protective immune response [19, 49, 50].

The loss of neutralization activity against chimeric CHIKV is in line with the finding that total IgG and anti-rE2 antibody titers correlate with the neutralizing titer of Asian serum (S6 Fig), suggesting that most of the neutralizing epitopes are on the E2 glycoprotein. The lack of correlation between anti-rE2 antibodies and neutralizing antibodies seen in ECSA serum could be due to the greater importance of conformational epitopes at E1-E2 sites, but we cannot exclude that it may reflect differences in potency/quality of the circulating antibodies due to the different timings of collection between the Asian and ECSA serum panels (S6 Fig). Correlation between serum neutralization titers and antibody binding titers has been reported in other viral infections such as dengue and influenza [51, 52], and is important for developing serological assays which are accurate correlates of protective immunity following infection or vaccination. Therefore, E2, while appropriate for serological assays to diagnose acute or past infection [53], may not be a suitable candidate for assays to measure protective immunity due to all CHIKV genotypes. Such assays are necessary for vaccine development.

Amino acid changes in key epitope regions, such as naturally occurring mutations or antigenic variation between different genotypes could affect surface charge distribution and electrostatic interactions between epitopes and antibodies, affect binding affinity and ultimately alter neutralizing capacity [14]. The E211K mutation in domain II of the E1 glycoprotein is a significant change of a negatively-charged to positively-charged amino acid, and this appears to enhance antibody binding and neutralization efficacy. During the recent Indian outbreak of ECSA CHIKV, the key amino acid change E1-K211E was shown to be under positive selection pressure [54], which may confer a selective advantage for virus dissemination and escape from the action of neutralization in humans. In addition, E211 is highly conserved in strains of the Asian genotype. Peptide-specific rabbit polyclonal antibody prepared against a short linear epitope (GDIQSRTPESKDVY, position 201–214) including 211K did not show neutralization activity (S7 Fig), suggesting that the neutralizing activity of immune sera targeting this amino acid is highly conformation-dependent. As for the E2 glycoprotein, I2T, H5N, G118S and S194G changes increased antibody binding and neutralization efficacy. All these amino acid changes are positioned within linear epitopes, which interacted with neutralizing antibodies. This was supported by a previous report of well-characterized human neutralizing monoclonal antibodies targeting epitopes that cluster around the LP24 and LP38 peptide regions in our study [43]. Notably, the linear epitope LP1 in our study is similar to E2EP3, a well-characterized key neutralizing linear epitope which has been suggested as a serology marker [26, 55], and LP1 demonstrated cross-reactivity with ECSA and Asian serum in our study. However, we found no effect of K252Q in antibody binding capacity in our cohort, although this was reported recently [14], and this could be due to differential immune responses in different populations. Other linear epitopes (LP19, LP47, LP56 and LP70) were identified in this study which had higher binding than LP1, and as all demonstrated binding to both ECSA and Asian sera, they may be potential candidates for diagnostic serological assays. Furthermore, antibodies against LP19 and LP47 demonstrated neutralizing characteristics which warrant further investigation as vaccine candidates (S8 Fig).

It was interesting that the Asian serum had greater neutralizing capacity against the heterologous ECSA isolates. The previously reported human CHIKV monoclonal antibodies 5F10 and 8B10 had a broad neutralization activity against isolates of the ECSA and West African genotypes, but were also less potent against an Asian isolate from Indonesia [56]. Monkeys inoculated with a virus-like particle vaccine derived from the West African strain 37997 also developed better neutralizing activity to a heterologous ECSA strain LR2006 OPY-1 than to 37997, possibly due to better presentation of conserved epitopes by LR2006 OPY-1 [49]. ECSA and Asian CHIKV genotypes could have induced different immune mediator profiles; as shown in mice, infection with a Caribbean (Asian) strain was associated with a weaker pro-inflammatory Th1 and natural killer cell response and higher IgG1:IgG2c ratio compared to an ECSA CHIKV strain, resulting in less severe joint pathology [57, 58]. Different CHIKV viruses may also trigger differential regulation of key innate immune responses such as TLR3 [59], which plays an important role in shaping subsequent neutralizing capacity [60]. Further studies are needed to understand how differentially-induced immune mediators modulate the properties of circulating serum antibodies.

Two amino acids in LP1 (2T, 5N) of the ECSA virus are critical for binding and neutralization activity, and this further highlights the fact that sequence variation could impact vaccine development. The rabbit polyclonal antibody targeting the linear neutralizing epitope LP1 from the ECSA virus showed reduced cross-neutralization against the Asian genotype, and unexpectedly, rabbit anti-LP1A poorly neutralized the homotypic CAR Asian virus, despite immunization of 4 rabbits. The linear neutralizing epitope LP1A from the Asian virus was not recognized by the ECSA sera. However, clearly there are preexisting antibodies against LP1 and LP1A in the Asian sera. LP47, another linear neutralizing epitope in humans (S8 Fig), which has a sequence that is conserved in both genotypes, did not induce any functional neutralizing antibodies in rabbits despite a similar immunization approach. Future studies will be required to address these apparent underlying differences of neutralizing antibody production from either natural infection or immunization. Nevertheless, our findings indicate that the choice of virus strain for vaccines could impact the spectrum and efficacy of protection across genotypes. For antibody therapy of CHIKV, monoclonal antibodies should retain high potency against a broad diversity of CHIKV isolates [43].

In conclusion, immune serum from humans infected with CHIKV of either ECSA or Asian genotypes showed differences in neutralization and binding capacities. Our findings are relevant to current outbreaks with co-circulating genotypes and provide insights into antibody-mediated immunity resulting from infections with CHIKV of different genotypes.

## Supporting Information

S1 Fig Seroneutralization of ECSA sera panel against ECSA and Asian CHIKV genotypes. Representative acquired immunofluorescence microscopic images of pooled serum at dilutions of 1:100, 1:400, and 1:1600, and virus control against clinical CHIKV isolates MY/08/065 (ECSA) and MY/06/37348 (Asian). Each image contains 9 combined fields within a well (96-well format). Objective magnification: 5X.
(PDF)

S2 Fig Seroneutralization of Asian sera panel against ECSA and Asian CHIKV genotypes. Representative acquired immunofluorescence microscopic images of pooled serum at dilutions of 1:100, 1:400, and 1:1600, and virus control against clinical CHIKV isolates MY/08/065 (ECSA) and MY/06/37348 (Asian). Each image contains 9 combined fields within a well (96-well format). Objective magnification: 5X.
(PDF)

S3 Fig Comparison of neutralizing capacity of heat-inactivated intact sera and DTT-treated sera. DTT-treated sera (containing IgG only) have similar neutralizing capacity to intact sera, which have a mixture of IgG and IgM. All sera were assayed up to 1:6400 dilution. The neutralization data was based on experiments performed against ECSA CHIKV (strain MY/08/065). Data are presented as means ± SEM; *n =* 23 for ECSA sera, *n =* 40 for Asian sera.
(PDF)

S4 Fig Seroneutralization of CHIKV immune individuals against Semliki Forest virus (SFV). (A) Seroneutralization was performed against SFV at 1:25 and 1:100 serum dilutions using pooled sera. Data are expressed as percentages of infectivity over infection control, and are presented as means ± SD from 3 independent experiments. **P< 0.01, ***P<0.001, Mann-Whitney U test relative to virus control. (B) Representative acquired immunofluorescence microscopic images of pooled serum at dilutions of 1:25 or 1:100 and virus control against SFV rescued from icDNA SFV6. Objective magnification: 10X.
(PDF)

S5 Fig Identification of amino acids on E2 which increased the antibody binding capacity. (A) Schematic representation of the E2 glycoprotein with the numbers indicating the amino acid positions of the glycoprotein and its domain proteins. The amino acid differences between E2 glycoproteins of ECSA (MY/08/065, ICRES1) and Asian (MY/06/37348, CAR) strains were tabulated and mapped (from amino acids 1–362). Amino acid differences within a genotype are underlined. Amino acid changes which fall within the identified linear epitopes are color-coded. (B) Immunoblotting was performed against recombinant E2 glycoproteins under reducing conditions, with each named amino acid change from the Asian to the ECSA sequence introduced independently. Mouse anti-His was used as a control. Site-directed mutagenesis was not performed for amino acid positions 312, 317 and 318 as these are predicted not to be exposed on the protein surface.
(PDF)

S6 Fig High titer of CHIKV-specific antibodies in Asian sera correlated with high antibody protection. The relationships between NT_50_ and antibody titers against (A) MY/08/065 and (B) recombinant E2 glycoprotein were assessed. Spearman’s rank correlation coefficients (ρ) and *P*-values are shown. *ns*, not significant.
(PDF)

S7 Fig Neutralization of CHIKV with peptide-specific rabbit polyclonal antibodies targeting the E1 glycoprotein. Two antibodies were prepared commercially; anti-E1DII, which targets a linear epitope of E1 (GDIQSRTPESKDVY, position 201–214), and anti-LP1, which targets a linear epitope of E2 (STKDNFNVYKATRPY, position 1–15). Seroneutralization was performed at 25μg/ml against ICRES1. Data are presented as means ± SD from 2 independent experiments, run in triplicate. ** *P*<0.01, Mann-Whitney U test relative to virus control.
(PDF)

S8 Fig Functional characterization of high linear epitope responders on the E2 glycoprotein. (A) Schematic diagram of the E2 protein showing the positions of the four mapped epitopes (LP19, yellow; LP47, pink; LP56, orange; LP70, sandy brown) which have higher OD relative to LP1. The numbers refer to the amino acid positions demarcating the E2 domains. N, N-link; C.arch, central arch. (B) Competitive peptide blocking assay was performed at 1:100 dilution with either pooled ECSA or Asian sera against ICRES1 at an MOI of 1. Sera blocked by LP19v and LP47 resulted in increases in infectivity. LP19v is a soluble peptide without cysteine residues at N- and C-terminuses of LP19. Data are expressed as percentages of infectivity of an infection control, and are presented as means ± SD from 2 independent experiments, run in triplicate. **P< 0.01, Mann-Whitney U test, relative to unblocked control. (C) The color-coded mapped neutralizing epitopes (LP19 and LP47) are localized on the structural E1-E2 heterodimer complex (based on PDB 3J2W).
(PDF)

S1 Table Primers used for mutagenesis of CHIKV E1 and E2 proteins and CHIKV infectious clones. (DOCX)

S2 Table Virus rescues after electroporation of DNA-launched icDNA CHIKV. (DOCX)

S3 Table Primers used for construction of expression cassettes. (DOCX)

S4 Table The fifty-nine overlapping peptides used for the peptide-based ELISA cover the CHIKV E2 glycoprotein sequence from amino acids 1 to 362, based on the CHIKV MY/08/065 sequence (accession no. FN295485). (DOCX)

S1 Text Supplementary materials and methods. (DOCX)
