# Supplementary figures and images for: Antigenic Variation of East/Central/South African and Asian Chikungunya Virus Genotypes in Neutralization by Immune Sera
Source: PLoS Negl Trop Dis. 2016 Aug 29;10(8):e0004960. doi: 10.1371/journal.pntd.0004960 (PMC5003353; doi:10.1371/journal.pntd.0004960)

**Pooled**  
**ECSA sera**

**MY/08/065 (ECSA)**

**MY/06/37348 (Asian)**

**1:100  
dilution**

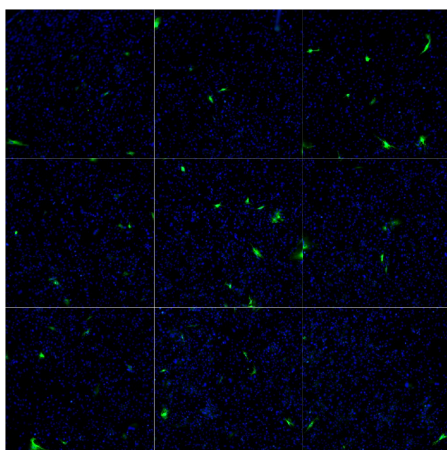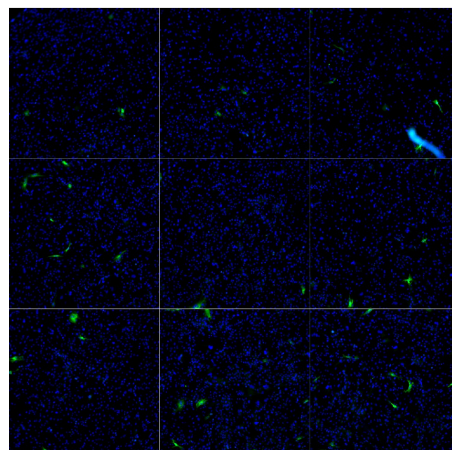

**1:400  
dilution**

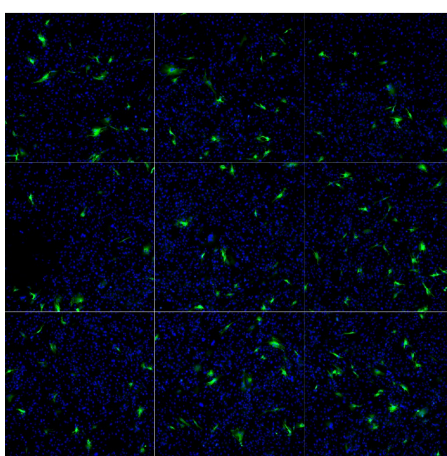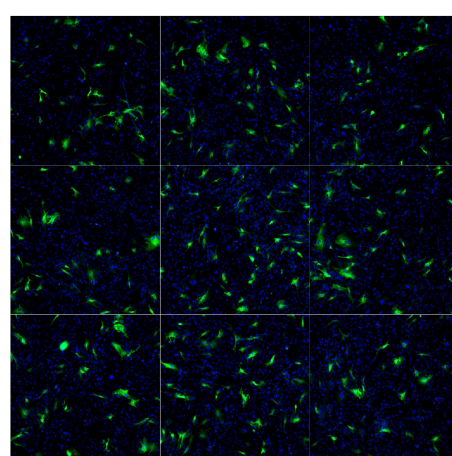

**1:1600  
dilution**

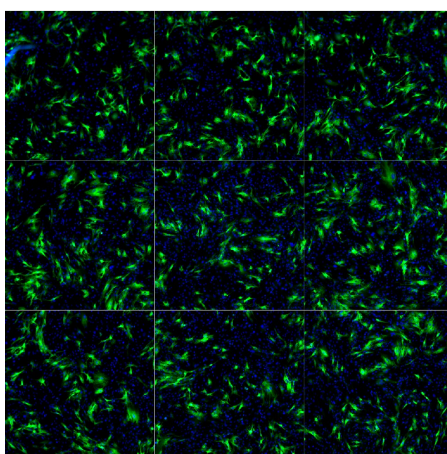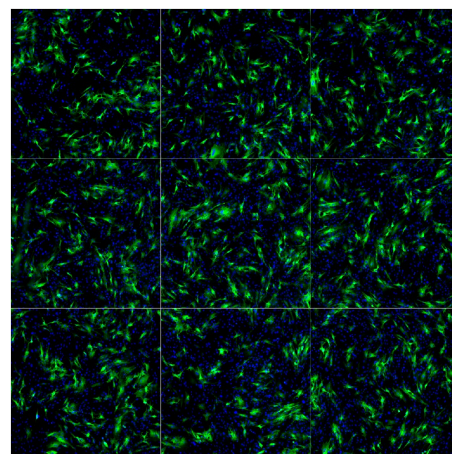

**Virus  
control**

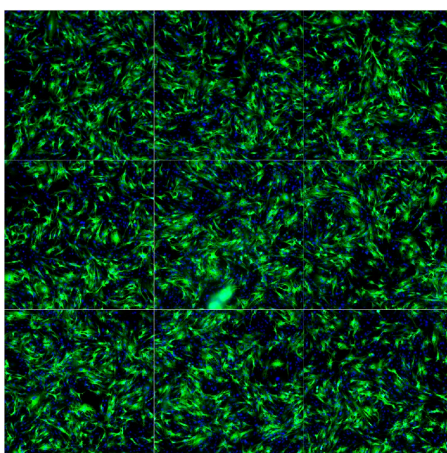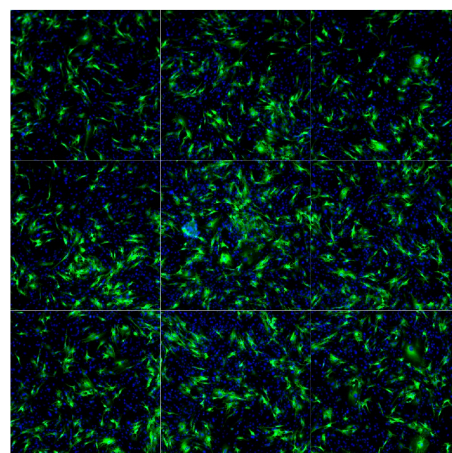

Supplement: S1 Fig — Representative acquired immunofluorescence microscopic images of pooled serum at dilutions of 1:100, 1:400, and 1:1600, and virus control against clinical CHIKV isolates MY/08/065 (ECSA) and MY/06/37348 (Asian). Each image contains 9 combined fields within a well (96-well format). Objective magnification: 5X. (PDF) [file pntd.0004960.s001.pdf]

**Pooled**  
**Asian sera**

**MY/08/065 (ECSA)**

**MY/06/37348 (Asian)**

**1:100  
dilution**

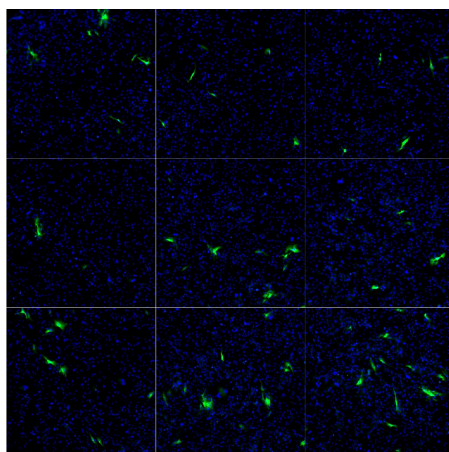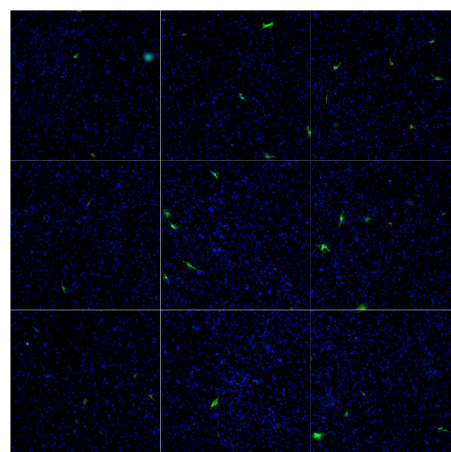

**1:400  
dilution**

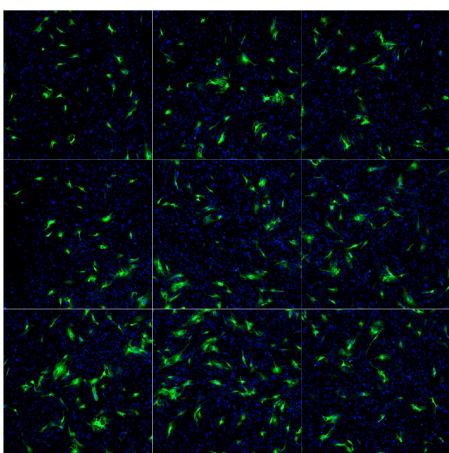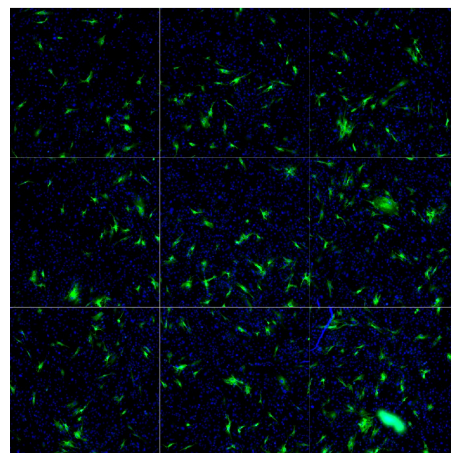

**1:1600  
dilution**

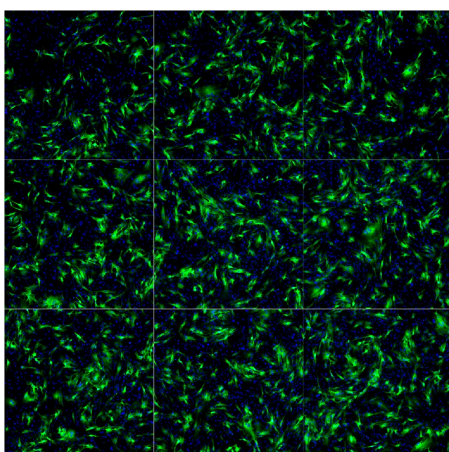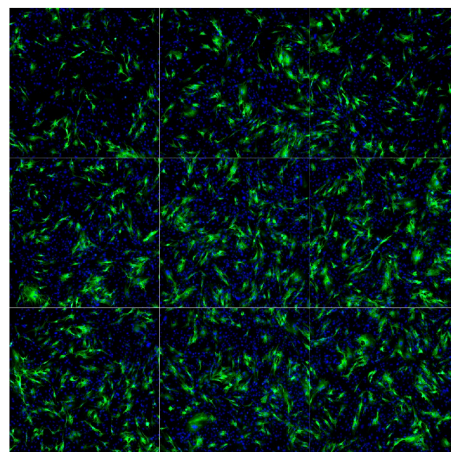

**Virus  
control**

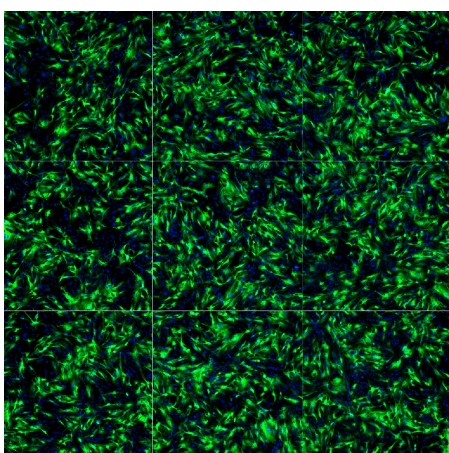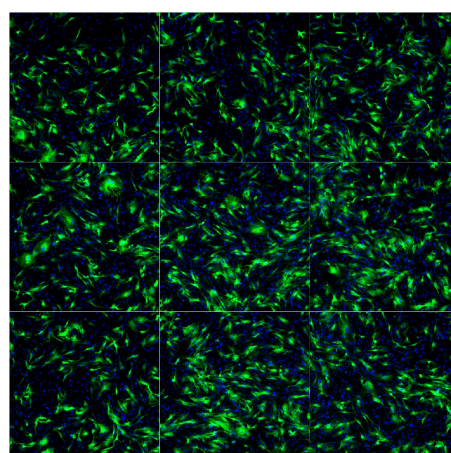

Supplement: S2 Fig — Representative acquired immunofluorescence microscopic images of pooled serum at dilutions of 1:100, 1:400, and 1:1600, and virus control against clinical CHIKV isolates MY/08/065 (ECSA) and MY/06/37348 (Asian). Each image contains 9 combined fields within a well (96-well format). Objective magnification: 5X. (PDF) [file pntd.0004960.s002.pdf]

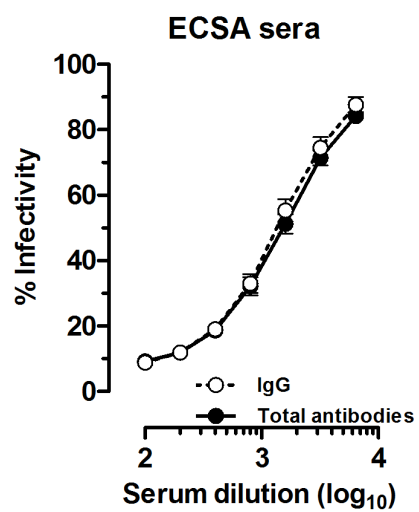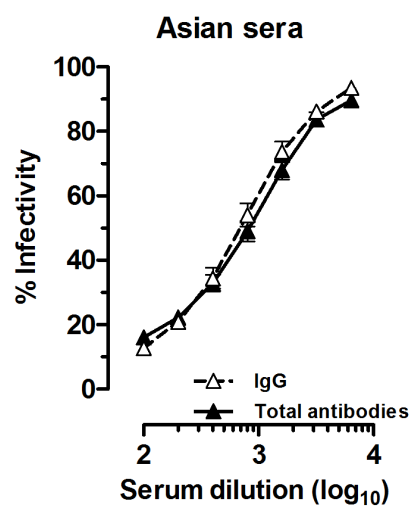

Supplement: S3 Fig — DTT-treated sera (containing IgG only) have similar neutralizing capacity to intact sera, which have a mixture of IgG and IgM. All sera were assayed up to 1:6400 dilution. The neutralization data was based on experiments performed against ECSA CHIKV (strain MY/08/065). Data are presented as means ± SEM; n = 23 for ECSA sera, n = 40 for Asian sera. (PDF) [file pntd.0004960.s003.pdf]

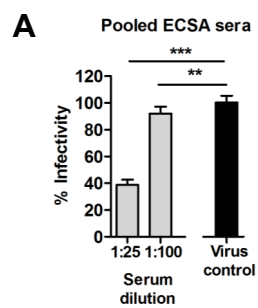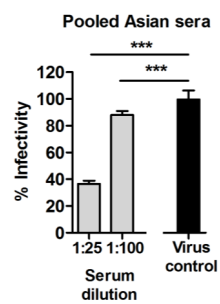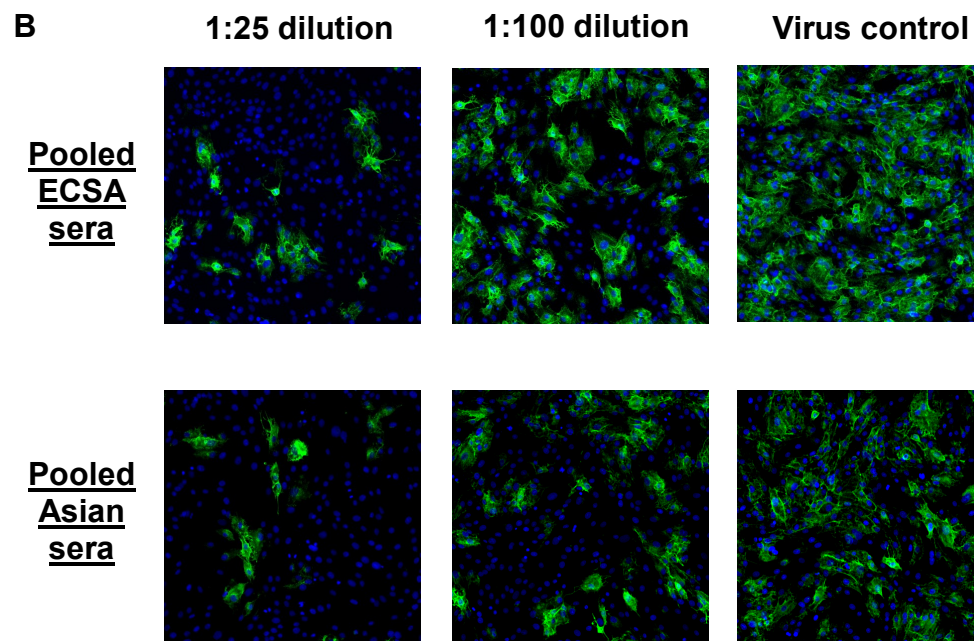

Supplement: S4 Fig — (A) Seroneutralization was performed against SFV at 1:25 and 1:100 serum dilutions using pooled sera. Data are expressed as percentages of infectivity over infection control, and are presented as means ± SD from 3 independent experiments. **P< 0.01, ***P<0.001, Mann-Whitney U test relative to virus control. (B) Representative acquired immunofluorescence microscopic images of pooled serum at dilutions of 1:25 or 1:100 and virus control against SFV rescued from icDNA SFV6. Objective magnification: 10X. (PDF) [file pntd.0004960.s004.pdf]

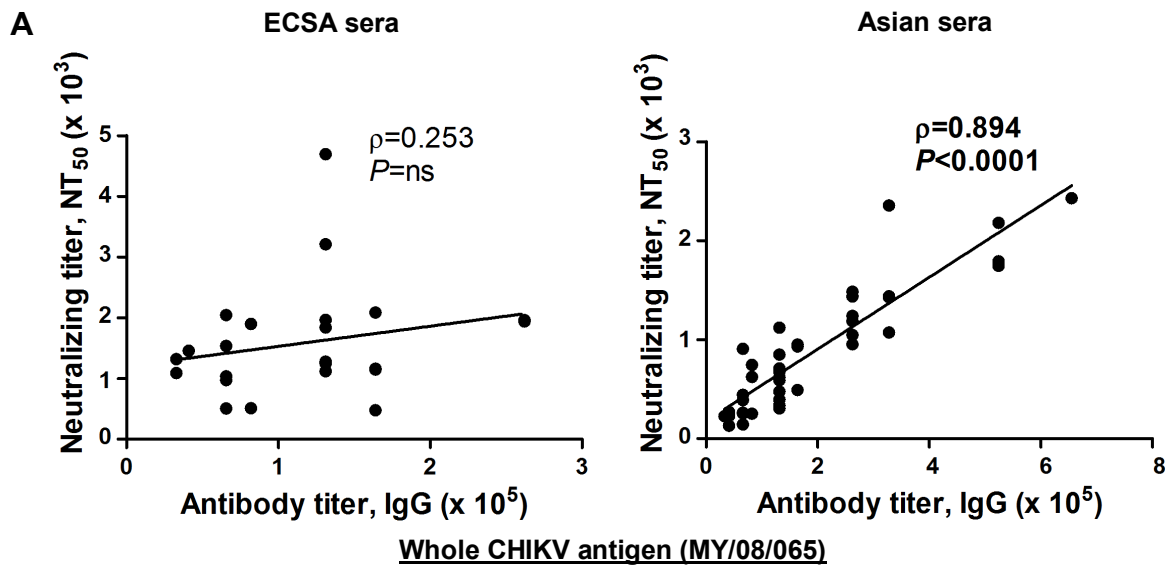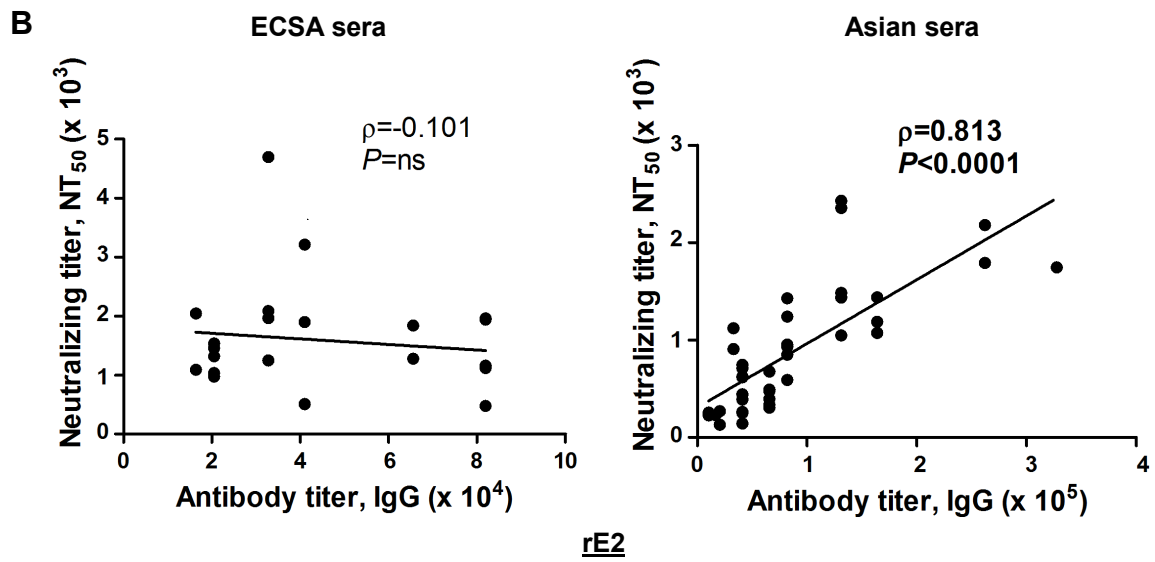

Supplement: S6 Fig — The relationships between NT50 and antibody titers against (A) MY/08/065 and (B) recombinant E2 glycoprotein were assessed. Spearman’s rank correlation coefficients (ρ) and P-values are shown. ns, not significant. (PDF) [file pntd.0004960.s006.pdf]

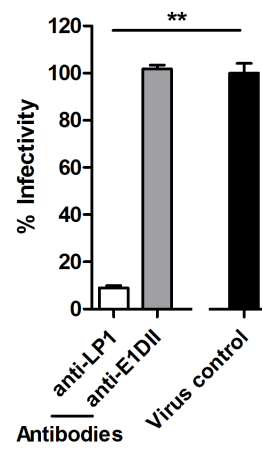

Supplement: S7 Fig — Two antibodies were prepared commercially; anti-E1DII, which targets a linear epitope of E1 (GDIQSRTPESKDVY, position 201–214), and anti-LP1, which targets a linear epitope of E2 (STKDNFNVYKATRPY, position 1–15). Seroneutralization was performed at 25μg/ml against ICRES1. Data are presented as means ± SD from 2 independent experiments, run in triplicate. ** P<0.01, Mann-Whitney U test relative to virus control. (PDF) [file pntd.0004960.s007.pdf]

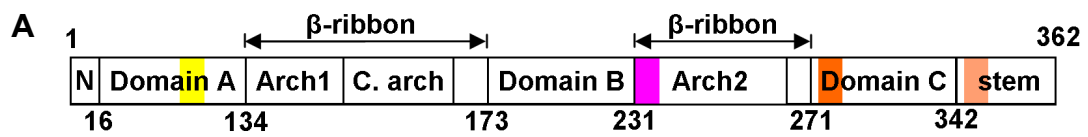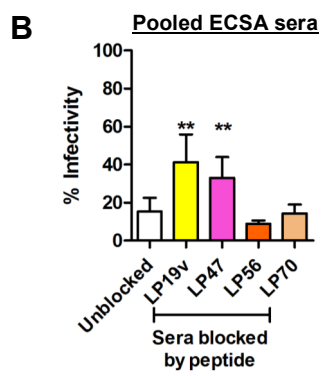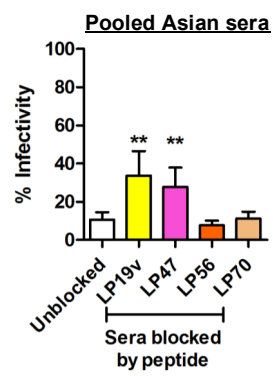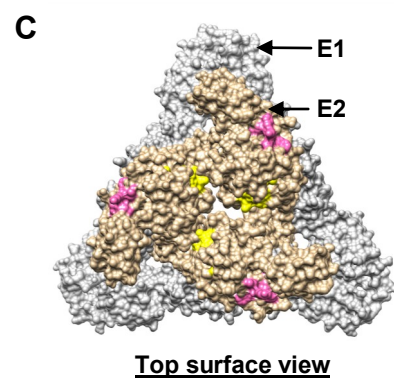

Supplement: S8 Fig — (A) Schematic diagram of the E2 protein showing the positions of the four mapped epitopes (LP19, yellow; LP47, pink; LP56, orange; LP70, sandy brown) which have higher OD relative to LP1. The numbers refer to the amino acid positions demarcating the E2 domains. N, N-link; C.arch, central arch. (B) Competitive peptide blocking assay was performed at 1:100 dilution with either pooled ECSA or Asian sera against ICRES1 at an MOI of 1. Sera blocked by LP19v and LP47 resulted in increases in infectivity. LP19v is a soluble peptide without cysteine residues at N- and C-terminuses of LP19. Data are expressed as percentages of infectivity of an infection control, and are presented as means ± SD from 2 independent experiments, run in triplicate. **P< 0.01, Mann-Whitney U test, relative to unblocked control. (C) The color-coded mapped neutralizing epitopes (LP19 and LP47) are localized on the structural E1-E2 heterodimer complex (based on PDB 3J2W). (PDF) [file pntd.0004960.s008.pdf]
