# Supplementary material for: Antigenic Variation of East/Central/South African and Asian Chikungunya Virus Genotypes in Neutralization by Immune Sera
Source: PLoS Negl Trop Dis. 2016 Aug 29;10(8):e0004960. doi: 10.1371/journal.pntd.0004960 (PMC5003353; doi:10.1371/journal.pntd.0004960)

A

ICRES1 (ECSA)	T	N	S	K	V	T	G	G	N	L	K	I	M	V	V
MY/08/065 (ECSA)	T	N	S	K	V	T	G	G	N	L	Q	I	M	V	V
Amino acid position	2	5	118	149	157	164	194	205	207	248	252	255	312	317	318
MY/06/37348 (Asian)	I	H	G	R	A	A	S	D	S	S	K	V	T	I	R
CAR (Asian)	I	H	G	R	A	A	S	D	S	F	K	V	T	I	R

B

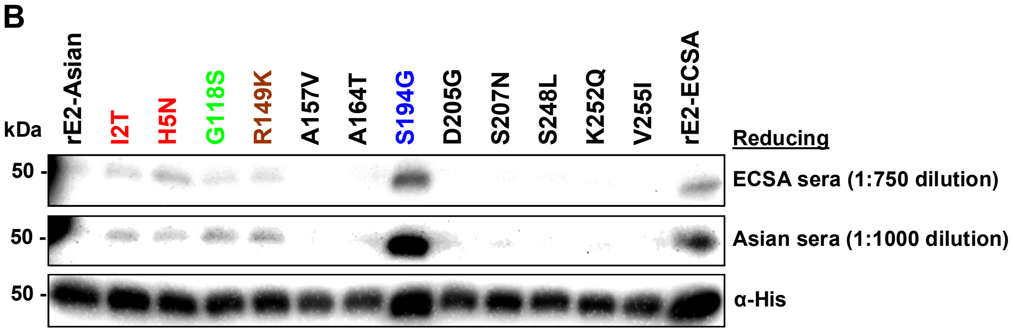

Supplement: S5 Fig — (A) Schematic representation of the E2 glycoprotein with the numbers indicating the amino acid positions of the glycoprotein and its domain proteins. The amino acid differences between E2 glycoproteins of ECSA (MY/08/065, ICRES1) and Asian (MY/06/37348, CAR) strains were tabulated and mapped (from amino acids 1–362). Amino acid differences within a genotype are underlined. Amino acid changes which fall within the identified linear epitopes are color-coded. (B) Immunoblotting was performed against recombinant E2 glycoproteins under reducing conditions, with each named amino acid change from the Asian to the ECSA sequence introduced independently. Mouse anti-His was used as a control. Site-directed mutagenesis was not performed for amino acid positions 312, 317 and 318 as these are predicted not to be exposed on the protein surface. (PDF) [file pntd.0004960.s005.pdf]
