# Supplementary material for: Antigenic Variation of East/Central/South African and Asian Chikungunya Virus Genotypes in Neutralization by Immune Sera
Source: PLoS Negl Trop Dis. 2016 Aug 29;10(8):e0004960. doi: 10.1371/journal.pntd.0004960 (PMC5003353; doi:10.1371/journal.pntd.0004960)
Supplement: S1 Table — (DOCX) [file pntd.0004960.s009.docx]

**S1 Table**

Primers used for mutagenesis of CHIKV E1 and E2 proteins and CHIKV infectious clones.

| **Mutation** | **5’-Sequence-3’** | | **Template** | | |
| --- | --- | --- | --- | --- | --- |
|  |  |  | **rE2-E1-Asian (antibody binding)** | **rE2-Asian**  **(antibody binding)** | **CAR**  **(infectious clone)** |
| E1-S72N | F  R | gcagagtgtaaggacaagaacctacctgattacagc  gctgtaatcaggtaggttcttgtccttacactctgc | ✓ |  |  |
| E1-T98A | F  R | cctactgcttctgcgacgccgaaaatacgcaattg  caattgcgtattttcggcgtcgcagaagcagtagg | ✓ |  |  |
| E1-A145T | F  R | cgtttgcataagcagttacagtgatattatttccttggtaaagg  cctttaccaaggaaataatatcactgtaactgcttatgcaaacg | ✓ |  | ✓ |
| E1-E211K | F  R | cgcacgcctgagagcaaagacgtctatgcta  tagcatagacgtctttgctctcaggcgtgcg | ✓ |  | ✓ |
| E1-S225A | F  R | ccgtacccgcggccggtctctgcag  ctgcagagaccggccgcgggtacgg | ✓ |  |  |
| E1-A226V | F  R | cagagaccgtccgtgggtacggtgcac  gtgcaccgtacccacggacggtctctg | ✓ |  | ✓ |
| E1-M269V | F  R | cccggtaagagcggtgaactgcgccgt  acggcgcagttcaccgctcttaccggg | ✓ |  | ✓ |
| E2-I2T | F  R | gctcgcggatcctagtactaaggaccacttcaatg  cattgaagtggtccttagtactaggatccgcgagc |  | ✓ |  |
| E2-H5N | F  R | cggatcctagtattaaggacaacttcaatgtctataaagcc  ggctttatagacattgaagttgtccttaatactaggatccg |  | ✓ | ✓ |
| E2-G118S | F  R | cggtggggttcactgatagtagaaagatcagtcac  gtgactgatctttctactatcagtgaaccccaccg |  | ✓ | ✓ |
| E2-R149K | F  R | cgaccgcagcacggtaaggaactaccttgc  gcaaggtagttccttaccgtgctgcggtcg |  | ✓ | ✓ |
| E2-A157V | F  R | gcagcacgtacgtgcagagcaccgc  gcggtgctctgcacgtacgtgctgc |  | ✓ |  |
| E2-A164T | F  R | gcagagcaccgctgcaactaccgaggagat  atctcctcggtagttgcagcggtgctctgc |  | ✓ |  |
| E2-S194G | F  R | gtaaagatcacagtcaatggtcagacggtgcggtac  gtaccgcaccgtctgaccattgactgtgatctttac |  | ✓ | ✓ |
| E2-S207N | F  R | agtgcaattgtggtgactcaaatgaaggattaaccactac  gtagtggttaatccttcatttgagtcaccacaattgcact |  | ✓ |  |
| E2-S248L | F  R | tccggtcccctaattcagcattgcgcgggac  gtcccgcgcaatgctgaattaggggaccgga |  | ✓ |  |
| E2-K252Q | F  R | GGAATGTGAACTTTTCCCTGCCGGTCCCCGGATTCAG  CTGAATCCGGGGACCGGCAGGGAAAAGTTCACATTCC |  | ✓ |  |
| E2-V255I | F  R | ccggggaccggaaaggaaaaattcacattccatttc  gaaatggaatgtgaatttttcctttccggtccccgg |  | ✓ |  |
| E2-I2T | F  R | gccagcggcgcagtactaaggaccacttcaa  ttgaagtggtccttagtactgcgccgctggc |  |  | ✓ |
| E2-I2T-H5N | F  R | cggcgcagtactaaggacaacttcaatgtctataaag  ctttatagacattgaagttgtccttagtactgcgccg |  |  | ✓ |
