# Supplementary material for: Antigenic Variation of East/Central/South African and Asian Chikungunya Virus Genotypes in Neutralization by Immune Sera
Source: PLoS Negl Trop Dis. 2016 Aug 29;10(8):e0004960. doi: 10.1371/journal.pntd.0004960 (PMC5003353; doi:10.1371/journal.pntd.0004960)
Supplement: S2 Table — (DOCX) [file pntd.0004960.s010.docx]

**S2 Table**

Virus rescues after electroporation of DNA-launched icDNA CHIKV.

| Backbone | Construct | Specific infectivity (pfu/1µg of DNA) | Virus titer (pfu/ml), P0,  24 hour | | Virus titer (pfu/ml),  P1,  24 hour |
| --- | --- | --- | --- | --- | --- |
| pCMV-ICRES1  (no extra subgenomic promoter and fluorescent marker) |  | 5.07 x 10^4^ | N.D. | |  |
| pCMV-ICRES1-2SG-zsGreen | ICRES1 | 3.73 x 10^4^ | 6.00 x 10^8^ | | 6.33 x 10^8^ |
|  | ICRES1  (sucrose cushion purified in TE buffer) | - | - | | 4.33 x 10^9^ |
|  | ICRES1-E1SFV | Non-viable | - | | - |
|  | ICRES1-E2SFV | 4.27 x 10^4^ | 1.00 x 10^7^ | | 8.00 x 10^7^ (48 h) |
|  | ICRES1-E1E2SFV | 5.60 x 10^4^ | 1.20 x 10^8^ | | 2.03 x 10^8^ (48 h) |
| pCMV-CAR (no extra subgenomic promoter and fluorescent marker) |  | 1.32 x 10^4^ | N.D. |  | |
| pCMV-CAR-2SG-zsGreen | CAR | 7.20 x 10^3^ | 2.03 x 10^7^ | | 1.37 x 10^8^ |
|  | CAR-E1-A145T | 2.31 x 10^4^ | 2.43 x 10^7^ | | 8.67 x 10^7^ |
|  | CAR-E1-E211K | 1.97 x 10^4^ | 1.17 x 10^7^ | | 2.47 x 10^8^ |
|  | CAR-E1-A226V | 9.33 x 10^3^ | 1.70 x 10^7^ | | 1.33 x 10^8^ |
|  | CAR-E1-M269V | 1.03 x 10^4^ | 1.60 x 10^7^ | | 1.37 x 10^8^ |
|  | CAR-E2-I2T | 6.40 x 10^3^ | 6.83 x 10^6^ | | 5.00 x 10^8^ |
|  | CAR-E2-H5N | 7.33 x 10^3^ | 3.50 x 10^6^ | | 5.00 x 10^8^ |
|  | CAR-E2-I2T-H5N | 9.60 x 10^3^ | 1.52 x 10^6^ | | 2.28 x 10^8^ |
|  | CAR-E2-G118S | 7.47 x 10^3^ | 1.00 x 10^6^ | | 5.00 x 10^8^ |
|  | CAR-E2-R149K | 1.01 x 10^4^ | 1.83 x 10^6^ | | 2.35 x 10^8^ |
|  | CAR-E2-S194G | 1.35 x 10^4^ | 2.10 x 10^7^ | | 1.23 x 10^8^ |
| pCMV-SFV6 | SFV6 | N.D. | 1.23 x 10^9^ | | 3.68 x 10^8^ |

N.D., not determined; ICRES1, CHIKV East/Central/South African genotype clone; CAR, CHIKV Asian genotype clone; SFV, Semliki Forest virus.
