# Supplementary material for: Antigenic Variation of East/Central/South African and Asian Chikungunya Virus Genotypes in Neutralization by Immune Sera
Source: PLoS Negl Trop Dis. 2016 Aug 29;10(8):e0004960. doi: 10.1371/journal.pntd.0004960 (PMC5003353; doi:10.1371/journal.pntd.0004960)
Supplement: S3 Table — (DOCX) [file pntd.0004960.s011.docx]

**S3 Table**

Primers used for construction of expression cassettes.

| Gene | Sequences with cloning sites integration (5’ to 3’) and underlined restriction sites (*Bam*H1 and *Not*1) ^1^ | | Vector |
| --- | --- | --- | --- |
| rE1-ECSA | E1-ECSA-F | GCGGATCCTTACGAACACGTAACAGTGATC | pIEX-5 |
|  | E1-ECSA-R | GCGCGGCCGCCTTCTGCACCCATGACATCGC |  |
| rE2-ECSA | E2-ECSA-F | GCGGATCCTAGCACCAAGGACAACTTCAAT | pIEX-5 |
|  | E2-ECSA-R | GCGCGGCCGCCAGCTCATAATAATACAGAAT |  |
| rE2-Asian | E2-A-F | GCGGATCCTAGTATTAAGGACCACTTCAAT | pIEX-5 |
|  | E2-A-R | Similar to E2-ECSA-R |  |
| Fusion E1-E2 glycoproteins   1. rE2-E1-Asian 2. Hybrid rE2_Asian_-E1_ECSA_ 3. Hybrid rE2_ECSA_-E1_Asian_ 4. rE2-E1-ECSA | First PCR  1. E2-A-F/E2-ECSA-F  2. E2-His8-R | ATGGTGATGGTGATGGTGGTGAGAACCGCCGCCCAGCTCATAATAATA | pIEX-5 |
|  | Second PCR  1. E1-His8-F  2. E1-A-R | CACCATCACCATCACCATCACGGCGGTGGTGGCTACGAACACGTAACA  Similar to E1-ECSA-R |  |

^1^ F, forward primer; R, reverse primer.
