# Supplementary material for: Antigenic Variation of East/Central/South African and Asian Chikungunya Virus Genotypes in Neutralization by Immune Sera
Source: PLoS Negl Trop Dis. 2016 Aug 29;10(8):e0004960. doi: 10.1371/journal.pntd.0004960 (PMC5003353; doi:10.1371/journal.pntd.0004960)
Supplement: S4 Table — (DOCX) [file pntd.0004960.s012.docx]

**S4 Table**

The fifty-nine overlapping peptides used for the peptide-based ELISA cover the CHIKV E2 glycoprotein sequence from amino acids 1 to 362, based on the CHIKV MY/08/065 sequence (accession no. FN295485).

| Binding region | Peptide annotation | Sequence |
| --- | --- | --- |
| N-link | LP1 | SGSGSTKDNFNVYKATRPY |
| Domain A | LP4 | SGSGLAHCPDCGEGHSCHS |
|  | LP5 | SGSGDCGEGHSCHSPVALE |
|  | LP6 | SGSGHSCHSPVALERIRNE |
|  | LP7 | SGSGPVALERIRNEATDGT |
|  | LP8 | SGSGRIRNEATDGTLKIQV |
|  | LP9 | SGSGATDGTLKIQVSLQIG |
|  | LP10 | SGSGLKIQVSLQIGIKTDD |
|  | LP11 | SGSGSLQIGIKTDDSHDWT |
|  | LP12 | SGSGIKTDDSHDWTKLRYM |
|  | LP13 | SGSGSHDWTKLRYMDNHMP |
|  | LP14 | SGSGKLRYMDNHMPADAER |
|  | LP15 | SGSGDNHMPADAERAGLFV |
|  | LP16 | SGSGADAERAGLFVRTSAP |
|  | LP17 | SGSGAGLFVRTSAPCTITG |
|  | LP18 | SGSGRTSAPCTITGTMGHF |
|  | LP19 | SGSGCTITGTMGHFILARC |
|  | LP20 | SGSGTMGHFILARCPKGET |
|  | LP21 | SGSGILARCPKGETLTVGF |
|  | LP22 | SGSGPKGETLTVGFTDSRK |
|  | LP23 | SGSGLTVGFTDSRKISHSC |
|  | LP24 | SGSGTDSRKISHSCTHPFH |
|  | LP25 | SGSGISHSCTHPFHHDPPV |
| β-ribbon (arch1) | LP28 | SGSGIGREKFHSRPQHGKE |
|  | LP29 | SGSGFHSRPQHGKELPCST |
|  | LP30 | SGSGQHGKELPCSTYVQST |
|  | LP31 | SGSGLPCSTYVQSTAATTE |
|  | LP32 | SGSGYVQSTAATTEEIEVH |
| Domain B | LP35 | SGSGMPPDTPDRTLMSQQS |
|  | LP36 | SGSGPDRTLMSQQSGNVKI |
|  | LP37 | SGSGMSQQSGNVKITVNGQ |
|  | LP38 | SGSGGNVKITVNGQTVRYK |
|  | LP39 | SGSGTVNGQTVRYKCNCGG |
|  | LP40 | SGSGTVRYKCNCGGSNEGL |
|  | LP41 | SGSGCNCGGSNEGLTTTDK |
|  | LP42 | SGSGSNEGLTTTDKVINNC |
|  | LP43 | SGSGTTTDKVINNCKVDQC |
|  | LP44 | SGSGVINNCKVDQCHAAVT |
| β-ribbon (arch 2) | LP47 | SGSGNHKKWQYNSPLVPRN |
|  | LP48 | SGSGQYNSPLVPRNAELGD |
|  | LP49 | SGSGLVPRNAELGDRQGKI |
|  | LP50 | SGSGAELGDRQGKIHIPFP |
|  | LP51 | SGSGRQGKIHIPFPLANVT |
|  | LP52 | SGSGHIPFPLANVTCRVPK |
| Domain C | LP55 | SGSGARNPTVTYGKNQVIM |
|  | LP56 | SGSGVTYGKNQVIMLLYPD |
|  | LP57 | SGSGNQVIMLLYPDHPTLL |
|  | LP58 | SGSGLLYPDHPTLLSYRNM |
|  | LP59 | SGSGHPTLLSYRNMGEEPN |
|  | LP60 | SGSGSYRNMGEEPNYQEEW |
|  | LP61 | SGSGGEEPNYQEEWVMHKK |
|  | LP62 | SGSGYQEEWVMHKKEVVLT |
|  | LP63 | SGSGVMHKKEVVLTVPTEG |
|  | LP64 | SGSGEVVLTVPTEGLEVTW |
|  | LP65 | SGSGVPTEGLEVTWGNNEP |
|  | LP66 | SGSGLEVTWGNNEPYKYWP |
| E2 stem | LP69 | SGSGQLSTNGTAHGHPHEI |
|  | LP70 | SGSGGTAHGHPHEIILYYY |
|  | LP71 | SGSGHPHEIILYYYELYPT |

Note: The N-terminus of each peptide was biotinylated and followed by SGSG residues as a spacer, and the C-terminus was amidated.
