# Supplementary material for: Antigenic Variation of East/Central/South African and Asian Chikungunya Virus Genotypes in Neutralization by Immune Sera
Source: PLoS Negl Trop Dis. 2016 Aug 29;10(8):e0004960. doi: 10.1371/journal.pntd.0004960 (PMC5003353; doi:10.1371/journal.pntd.0004960)
Supplement: S1 Text — (DOCX) [file pntd.0004960.s013.docx]

**S1 Text**

**Virus rescue from icDNA**

The plasmid vectors containing icDNA were used to transform XL-10 Gold (Agilent) cells by the calcium chloride method. Endotoxin-free plasmids were prepared with PureLink HiPure Plasmid Miniprep Kit (Life Technologies) in ultra-pure H_2_O (Life Technologies) and the yield was quantitated. Five micrograms of each plasmid was used to electroporate into 3.5-4 x 10^6^ BHK-21 cells using Gene Pulser Xcell electroporation systems (Bio-Rad) under the following conditions: 220V, 975µF, one pulse in a 4mm cuvette, with a total volume of 250µl of cell suspension accompanied with plasmid and 50µg of salmon sperm DNA carrier (Life Technologies) in 10% FBS GMEM (in the absence of penicillin/ streptomycin). After electroporation, the cells were immediately recovered with the addition of media. A small aliquot of electroporation mixture was transferred out for the infectious center assay and the rest was transferred into T-75cm^2^ flasks in 10% FBS GMEM. After incubation for 24 hours at 37°C, P0 rescued viruses stocks were harvested and titrated by plaque assay.

**Infectious center assay**

Fresh 10% FBS GMEM was added to the aliquot of electroporated BHK-21 cells, which was then serially ten-fold diluted and transferred to 6-well plates containing pre-seeded, uninfected BHK-21 cells (6 x 10^5^ cells per well). After 2-3 hours of incubation at 37°C, cells were overlaid with 2ml of plaque medium in each well (2% FBS GMEM containing 0.8% of carboxymethylcellulose). After 3-4 days incubation, the cells were fixed and stained with crystal violet. Plaques were counted and specific infectious virus rescue efficiency (“infectivity”) was expressed as the number of plaque-forming units per 1 µg of electroporated DNA. The specific infectivities of all constructs are tabulated in S2 Table.

**Plaque assay**

The clarified, rescued virus supernatant was serially diluted ten-fold in serum free MEM (containing 20mM HEPES, 0.2% BSA, 5mM L-glutamine) and transferred to 6-well plate containing pre-seeded BHK-21 cells (8 x 10^5^ cells per well). The plate was incubated for 1 hour at 37°C with intermittent rocking every 15 minutes. The cells were overlaid with 2ml of plaque medium and the plates were incubated for three days. The plates were fixed, stained and scored visually.

**Construction of cassette encoding for E1-E2 fusion protein**

Two separate PCR reactions were performed independently. The first amplification reaction of the E2 region (amino acids 1-362) and the second amplification reaction of the E1 region (amino acids 1-412) were performed for either Asian or ECSA genotypes using the primer sets in S3 Table. The reverse primer in the first reaction and forward primer in the second reaction have an integrated sequence corresponding to a short peptide linker and 8x His-tag. Both PCR reactions were cleaned up, and another round of overlapping PCR was performed by mixing both cleaned up amplicons in a reaction, with the forward primer from the first PCR reaction and the reverse primer from the second PCR reaction. The final amplicons were cleaned up and ligated into a pIEX-5 vector (Novagen) directionally at *BamH1* and *Not1* restriction sites.
